# Isolated, reduced diffusing capacity for carbon monoxide in the population – relations to symptoms, smoking and comorbidities

**DOI:** 10.1186/s12890-026-04662-9

**Published:** 2026-08-31

**Authors:** Viktor Hamrefors, Johan Westerbergh, Annelie Behndig, Anders Blomberg, John Brandberg, Anna Carlén, Arne Egesten, Emil Ekbom, Össur Ingi Emilsson, Maria J. Eriksson, Andreas Frølich, Ludger Grote, Christer Janson, Reza Karimi, Anna Moberg, Hans Lennart Persson, Ida Pesonen, Magnus Sköld, Karen Sörensen, Hanan Tanash, Kjell Torén, Lowie Vanfleteren, Per Wollmer, Andrei Malinovschi

**Affiliations:** 1 Department of Clinical Sciences, Clinical Research Centre, Lund University and Department of Internal Medicine, Skåne University Hospital, Box 50332, Malmö, 202 13 Sweden; 2 Uppsala Clinical Research Center, Uppsala University, Uppsala, Sweden; 3 Department of Public Health and Clinical Medicine, Umeå University, Umeå, Sweden; 4 Department of Radiology, Institute of Clinical Sciences, Sahlgrenska Academy at University of Gothenburg, Gothenburg, Sweden; 5 Department of Radiology, Region Västra Götaland, Sahlgrenska University Hospital, Gothenburg, Sweden; 6 Department of Health, Medicine and Caring Sciences, Linköping University, Linköping, Sweden; 7 Department of Clinical Physiology in Linköping, Region Östergötland, Linköping, Sweden; 8 Respiratory Medicine, Allergology, and Palliative Medicine, Department of Clinical Sciences Lund, Lund University and Skåne University Hospital, Lund, Sweden; 9 Department of Medical Sciences, Respiratory, Allergy and Sleep Research, Uppsala University, Uppsala, Sweden; 10 Faculty of Medicine, Reykjavik and Department of Respiratory Medicine, University of Iceland, Landspitali University Hospital, Reykjavik, Iceland; 11 Department of Molecular Medicine and Surgery, Karolinska Institute, Stockholm, Sweden; 12 Department of Clinical Physiology, Karolinska University Hospital, Stockholm, Sweden; 13 Department of Internal Medicine and Clinical Nutrition, Institute of Medicine, Sahlgrenska Academy, University of Gothenburg, Gothenburg, Sweden; 14 Division of Immunology and Respiratory Medicine, Department of Medicine Solna and Center for Molecular Medicine, Karolinska Institutet, Stockholm, Sweden; 15 Department of Respiratory Medicine and Allergy, Karolinska University Hospital, Stockholm, Sweden; 16 Department of Respiratory Medicine in Linköping, Linköping University, Linköping, Sweden; 17 Department of Diagnostics and Intervention, Umeå University, Umeå, Sweden; 18 Department of Medicine, Skåne University Hospital, Lund University, Lund, Sweden; 19 Section of Occupational and Environmental Medicine, School of Public Health and Community Medicine, Institute of Medicine, Sahlgrenska Academy, University of Gothenburg, Gothenburg, Sweden; 20 Department of Occupational and Environmental Medicine, Sahlgrenska University Hospital, Gothenburg, Sweden; 21 Department of Internal Medicine and Clinical Nutrition, COPD Center, Sahlgrenska Academy, University of Gothenburg, Gothenburg, Sweden; 22 Department of Translational Medicine, Lund University, Malmö, Sweden; 23 Department of Medical Sciences, Clinical Physiology, Uppsala University, Uppsala, Sweden

**Keywords:** Diffusion capacity for carbon monoxide, Emphysema, General population, Smoking, Lung function

## Abstract

**Background:**

Diffusing capacity for carbon monoxide (D_LCO_) is a measure of gas exchange in the lungs. Reduced D_LCO_ with normal spirometry is a common finding that is incompletely understood.

**Aim:**

To assess determinants of isolated, reduced D_LCO_ in an unselected population.

**Methods:**

The Swedish CArdioPulmonary bioImage Study (SCAPIS) is a multicentre population-based study of adults aged 50–64 years. The 5th percentile of D_LCO_ in healthy never smokers was used as the lower limit of normal (LLN). Using multivariable analysis, we studied factors associated with D_LCO_ < LLN in 24 261 subjects with normal spirometry, defined as FEV1, FVC and FEV1/FVC > LLN. Emphysema and interstitial lung abnormalities (ILA) were assessed through computed tomography, physical activity through accelerometry and other factors through questionnaires.

**Results:**

Isolated D_LCO_ < LLN (*n* = 1 472) was independently associated with emphysema (odds ratio and 95% confidence interval: 2.6 (2.2, 3.2)), lung disease other than asthma or COPD (1.8 (1.2, 2.8)), exertional chest pain (1.6 (1.3, 2.1)), smoking (per-10-pack-years) (1.6 (1.4, 1.9)), ILA (1.4 (1.2, 1.7)), breathing problems (1.4 (1.1, 1.8)), male sex (1.3 (1.1, 1.6)) and coughing (1.2 (1.1, 1.5)). D_LCO_ < LLN was less likely if subjects were older, had asthma or had higher BMI, haemoglobin, FVC or physical activity.

**Conclusion:**

Isolated, reduced D_LCO_ in a large middle-aged population cohort is associated with numerous previously known factors, including emphysema, ILA, smoking and self-reported lung disease. However, several symptoms, including exertional chest pain, breathing problems and coughing, are also independently associated with isolated reduced D_LCO_.

**Supplementary Information:**

The online version contains supplementary material available at https://doi.org/10.1186/s12890-026-04662-9.

## Introduction

Measurement of the diffusing capacity for carbon monoxide (D_LCO_) provides valuable insights into gas exchange in the lungs. However, impaired diffusion is far from the only cause of reduced D_LCO_. Measurement is also affected by the transfer of the inhaled gas mixture into airways and the alveolar space through bulk flow, mixing and diffusion of carbon monoxide (CO) in the alveolar ducts, air sacs and alveoli, transfer of CO across the alveolar-capillary barrier, by mixing and diffusion in the lung tissue and blood, and by diffusion across the red cell membrane and reaction with haemoglobin (Hb) [1]. It is difficult to determine the underlying pathology based solely on measurement of D_LCO_. If information on D_LCO_ is integrated with findings from spirometry, measurement of static lung volume or results of radiological examinations, the situation may be clarified. Furthermore, D_LCO_ can be affected by respiratory diseases, diseases affecting the heart (heart failure) [2] or pulmonary circulation (pulmonary arterial hypertension) [3], systemic rheumatic diseases (such as systemic lupus erythematosus [4]) or anaemia [5].

Significantly reduced D_LCO_ in combination with normal spirometry is not an uncommon finding [6], but is difficult to interpret. There are few studies on this clinical problem. Studies aiming to determine the underlying pathology have been retrospective [7, 8] and therefore likely to be fraught with selection bias, or were performed in selected groups, such as smokers [9, 10]. We are aware of only two studies in a population-based setting [6, 11]. The largest of these included 1,232 subjects, 145 of whom had abnormal D_LCO_ in the absence of airflow obstruction. Male sex and current smoking were the only characteristics significantly associated with low D_LCO_ and normal spirometry.

Thus, whereas there are several studies assessing factors associated with low D_LCO_ in clinical samples, the causes of isolated, reduced D_LCO_ in the general population remains incompletely known. In the current study, we aimed to test the hypothesis that certain factors, including self-reported symptoms, subclinical cardiovascular disease, smoking habits and specific radiological findings, would be associated with the finding of isolated, reduced D_LCO_ in middle-aged subjects from the general population. Additionally, we used interaction analyses to investigate whether these relations were affected by smoking habits.

## Methods

### Subjects

The current study was based on the population-based Swedish CArdioPulmonary bioImage Study (SCAPIS). The aim of SCAPIS was to investigate cardiopulmonary diseases in 30,000 subjects aged 50–64 years from the general population. Subjects were chosen randomly and invited by letter, as previously described in detail [12]. About half of the invited people agreed to participate and the study included 30,154 subjects in total. They were examined at one of the six screening centres between 2013 and 2018. The ethical review board in Umeå approved SCAPIS as a multicentre study (Dnr 2010–228-31 M), with subsequent approval of analysis of pulmonary function (Dnr 2017/14–31). The study adhered to the Declaration of Helsinki and all participants provided written informed consent.

**Fig. 1:**
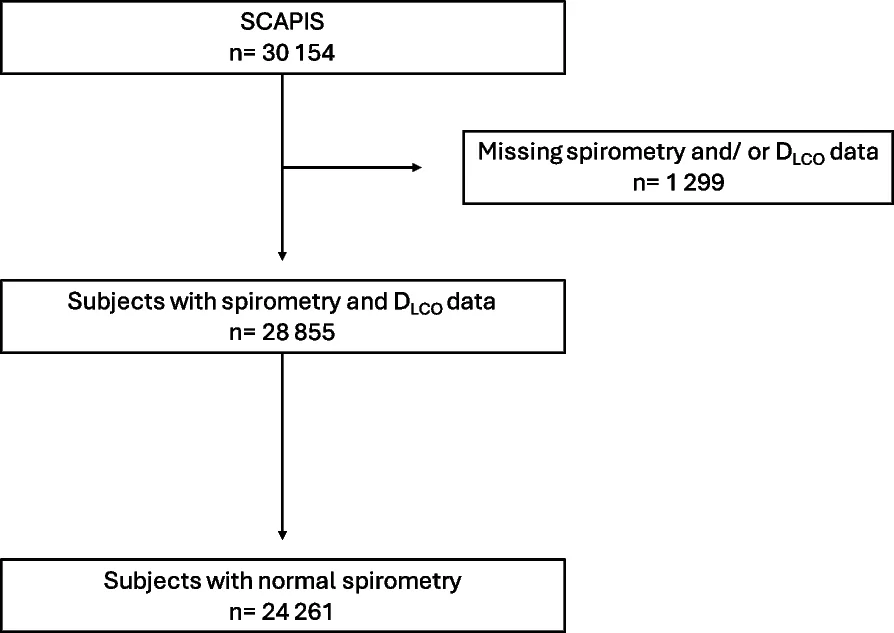
Selection of study participants. SCAPIS = The Swedish CArdioPulmonary bioImage Study. D_LCO_ = Diffusing capacity for carbon monoxide. Normal spirometry was defined as FEV_1_, FVC and FEV_1_/FVC ≥ LLN in SCAPIS [14]

The overlap between lung function impairment and emphysema in relation to smoking habits in the SCAPIS population has been recently described [13]. The current analyses focused only on participants with normal spirometry (*n* = 24,261) (Fig. 1).

### Anthropometric, self-reported and biochemical variables

Body mass index (BMI) was computed as body weight/squared height (kg/m^2^). An Omron M10-IT automatic device (www.omron.com) was used to measure systolic and diastolic blood pressure twice in each arm in the supine position, with a one-minute interval between measurements. In the analysis, we used the mean systolic and diastolic blood pressure from the arm with the highest mean systolic blood pressure.

Information on smoking, symptoms and any other diseases that had been diagnosed by a physician were self-reported. The symptom and comorbidity questionnaire items were obtained from the core SCAPIS questionnaire, and the exact wording used for each question can be found in the Supplemental Material 1 (Core Questions SCAPIS). Further details of all variables are available at https://www.scapis.org/portal/variables. We grouped the participants as current smokers, ex-smokers and never smokers. Physical activity was assessed using ActiGraph GT3X or GT3X + activity monitors [15], which measured physical activity for seven days in a row.

### Lung function and computed tomography imaging of lungs

The SCAPIS core protocol included only post-bronchodilator spirometry. Jaeger MasterScreen PFT (Carefusion, Hoechberg, Germany) was used to perform dynamic spirometry 15 min after giving 400 μg of salbutamol to a subject. Each examination was performed with the subject in the sitting position, wearing a nose clip. Forced expiratory volume in 1 s (FEV_1_), forced vital capacity (FVC) were measured in accordance with the American Thoracic Society and European Respiratory Society standards [16] and the FEV_1_/FVC ratio was calculated. We used the single-breath D_LCO_ test (Jaeger MasterScreen PFT) to measure CO uptake for assessing D_LCO_. Reference values for all spirometry indices and D_LCO_ were generated based on data for healthy never smokers from SCAPIS [14]. These reference values have been suggested to be more appropriate for assessing D_LCO_ in the SCAPIS population compared to The Global Lung Function Initiative (GLI) reference equations, as GLI underestimated impaired D_LCO_ in a SCAPIS interim analysis [17].

Computed tomography (CT) of lungs was performed using a dual-source CT scanner with a Stellar Detector (Somatom Definition Flash, Siemens, Forchheim, Germany). The CT scanners had the same software and hardware at all six sites. Radiologists did visual scoring of emphysema from the CT images, using a modified score sheet from the COPD Gene study (www.copdgene.org), as previously described [18]. A participant was considered to have interstitial lung abnormalities (ILA) if there were any cysts, ground-glass, reticular, bronchiectasis or honeycomb findings, in accordance with the definitions used in a recent SCAPIS publication [19].

### Assessment of atherosclerosis

The details of the assessment of atherosclerosis in SCAPIS were recently published [12]. Cardiac CT imaging was used to assess coronary calcium scores in accordance with Agaston and CT coronary angiography was used to detect any plaques in the coronary arteries, as previously described in detail [12].

### Statistics

#### Derived variables

Lower limit of normal (LLN): LLNs were calculated for D_LCO_ (uncorrected for haemoglobin levels), FEV_1_, FVC and FEV_1_/FVC using the SCAPIS reference equations [14]. A subject was considered to have normal spirometry if all three variables FEV_1_/FVC, FEV_1_ and FVC were ≥ LLN. If these requirements were not fulfilled, the subject was denoted as having ‘abnormal spirometry’.

#### Missing data

Of the 24 261 subjects with normal spirometry, 4 084 had missing data on one or more of the main variables, yielding 20 177 individuals with complete observations for the main variables. For the multivariable analysis, an imputed dataset was created with multivariate imputation performed using chained equations. The imputations were performed in R using the package *mice*. The number of imputations was set to 20.

#### Statistical methods

Descriptive statistics for main variables are presented as medians and interquartile ranges for continuous variables and as frequencies for categorical variables.

The characteristics of participants with normal spirometry are also presented stratified by D_LCO_ (normal D_LCO_ and D_LCO_ < LLN) and smoking status (never smoker, ex-smoker and current smoker).

The associations between the main variables and D_LCO_ were examined either through logistic regression using D_LCO_ < LLN or through linear regression using D_LCO_ as a proportion of the reference value. The univariable analysis was performed as a complete case analysis, whereas the primary multivariable analysis was performed on imputed data, with a complete case analysis as a secondary analysis. Continuous variables were fitted using splines with 4 knots. The multivariable model included the variables age, sex, BMI, height, any emphysema on CT, atherosclerosis in any coronary segment, ILA, Hb, FVC, average physical activity intensity in counts per minute, self-reported diagnoses (hypertension, chronic obstructive pulmonary disease (COPD), chronic bronchitis or emphysema, asthma, other lung disease, sleep apnoea), self-reported symptoms (coughing, phlegm, wheezing, breathing problems affecting daily life, chest pain when walking uphill), and smoking history (cigarette pack-years).

The results are presented in forest plots showing either the odds ratio (OR) or the effect on proportion of D_LCO_ between two binary levels or between the 25th (Q_1_) and 75th percentile (Q_3_) for continuous variables.

A stratified or interaction analysis was performed for the multivariable analysis estimating the effects in the three smoker subgroups: never smoker, ex-smoker and current smoker. The variable pack-years was removed from this model and the interaction between all variables and smoking status was included.

#### Statistical software

All analyses were made using R version 3.6.1.

## Results

### Study population characteristics

Subjects with reduced D_LCO_ reported more often cardiopulmonary diseases, current smoking and had slightly lower values of spirometry indices (Table 1, Supplementary Table 1). There was a higher proportion of subjects with normal spirometry and D_LCO_ < LLN among current smokers than among former and never smokers (16.8%, 5.8% and 4.1%, respectively). Among current smokers, those with D_LCO_ < LLN showed the highest prevalence of imaging abnormalities, reported more symptoms and reported a higher prevalence of comorbidities (Supplementary Table 2).

**Table 1:** Clinical characteristics

	**Normal spirometry and D**_**LCO**_** ≥ LLN**	**Normal spirometry and D**_**LCO**_** < LLN**	**All subjects**
	***N* = 22789**	***N* = 1472**	***N* = 24261**
Basic data			
Age, years	57.2 (53.5–61.0)	57.2 (53.4–61.2)	57.2 (53.5–61.0)
Sex: Female	11796 (51.8%)	826 (56.1%)	12622 (52.0%)
Male	10993 (48.2%)	646 (43.9%)	11639 (48.0%)
Anthropometry			
BMI, kg/m^2^	26.3 (23.9–29.2)	26.1 (23.6–29.1)	26.3 (23.9–29.2)
Height, m	172.0 (165.0–179.0)	171.0 (164.8–178.0)	172.0 (165.0–179.0)
Imaging			
Any emphysema	792 (3.5%) [299]	178 (12.3%) [25]	970 (4.1%) [324]
Atherosclerosis in any coronary segment	8714 (41.3%) [1702]	594 (45.8%) [176]	9308 (41.6%) [1878]
ILA	2290 (10.2%) [328]	233 (16.2%) [30]	2523 (10.6%) [358]
Biochemistry			
Hb g/L	141.0 (133.0–150.0) [137]	138.0 (130.0–147.0) [10]	141.0 (133.0–149.0) [147]
Lung function			
Chronic airflow limitation	0 (0.0%)	0 (0.0%)	0 (0%)
D_LCO_, mmol min^−1^ kPa^−1^	8.6 (7.3–10.1)	6.1 (5.5–7.2)	8.4 (7.2–10.0)
FEV_1_, L	3.3 (2.8–3.9)	3.0 (2.6–3.6)	3.3 (2.8–3.9)
FVC, L	4.2 (3.5–5.0)	3.9 (3.3–4.6)	4.2 (3.5–5.0)
FEV_1_/FVC	0.79 (0.76–0.82)	0.79 (0.75–0.82)	0.79 (0.76–0.82)
D_LCO,_ % of predicted*	99.6 (91.8–108.5)	75.6 (71.7–78.0)	98.6 (90.1–107.7)
D_LCO_, z-score	0.0 (−0.6 – 0.6)	−2.0 (−2.4 – −1.8)	−0.1 (−0.8 – 0.6)
FEV_1_,% of predicted*	100.8 (93.7–108.3)	94.2 (88.5–101.2)	100.4 (93.2–108.0)
FVC, % of predicted*	100.7 (93.6–108.3)	94.9 (89.0–102.0)	100.4 (93.3–107.9)
FEV_1_/FVC, % of predicted*	99.8 (95.7–103.6)	99.0 (94.7–103.2)	99.7 (95.6–103.6)
VA, % of predicted**	100.6 (94.0–107.5)	92.8 (87.0–99.5)	100.2 (93.5–107.1)
VA, z-score	0.1 (−0.5 – 0.7)	−0.6 (−1.2 – 0.0)	0.0 (−0.6 – 0.6)
Physical activity			
Average physical activity intensity, cpm	662.2 (538.2–800.5) [780]	601.8 (480.8–729.8) [71]	658.7 (535.0–796.3) [851]
Comorbidities			
Hypertension	4734 (21.5%) [753]	352 (25.1%) [71]	5086 (21.7%) [824]
COPD, CB or emphysema	80 (0.4%) [795]	18 (1.3%) [81]	98 (0.4%) [876]
Asthma	1547 (7.0%) [795]	75 (5.4%) [81]	1622 (6.9%) [876]
Other lung disease	216 (1.0%) [795]	31 (2.2%) [81]	247 (1.1%) [876]
Sleep apnoea	928 (4.2%) [795]	56 (4.0%) [81]	984 (4.2%) [876]
Symptoms			
Coughing	3652 (16.5%) [686]	331 (23.7%) [74]	3983 (16.9%) [760]
Phlegm	2050 (9.3%) [765]	195 (14.0%) [76]	2245 (9.6%) [841]
Wheezing	1112 (5.1%) [803]	121 (8.7%) [84]	1233 (5.3%) [887]
Breathing problems affecting daily life	866 (3.9%) [699]	84 (6.0%) [77]	950 (4.0%) [776]
Chest pain walking uphill	641 (2.9%) [690]	95 (6.8%) [70]	736 (3.1%) [760]
Smoking			
Smoking data			
Never smoker	11841 (53.6%) [705]	501 (35.0%) [42]	12342 (52.5%) [747]
Ex-smoker	8093 (36.6%)	496 (34.7%)	8589 (36.5%)
Current smoker	2150 (9.7%)	433 (30.3%)	2583 (11.0%)
Pack years	0.0 (0.0–9.5)	7.5 (0.0–22.0)	0.0 (0.0–10.0)

Data shown as number (percent) for dichotomous variables and as median (Q1–Q3) for continuous variables

[ ] denotes the number of missing values

^*^based on the SCAPIS reference equations [14]

^**^the reference equation for VA is shown in Supplementary Fig. 7

*BMI* body mass index, *COPD* chronic obstructive pulmonary disease, *CB* chronic bronchitis, *cpm* counts per minute, *D*_*LCO*_ diffusing capacity for carbon monoxide, *FEV*_*1*_ forced expiratory volume in 1 s, *FVC* forced vital capacity, *ILA* interstitial lung abnormalities, *LLN* lower limit of normal

### Factors associated with isolated, reduced DLCO

**Fig. 2:**
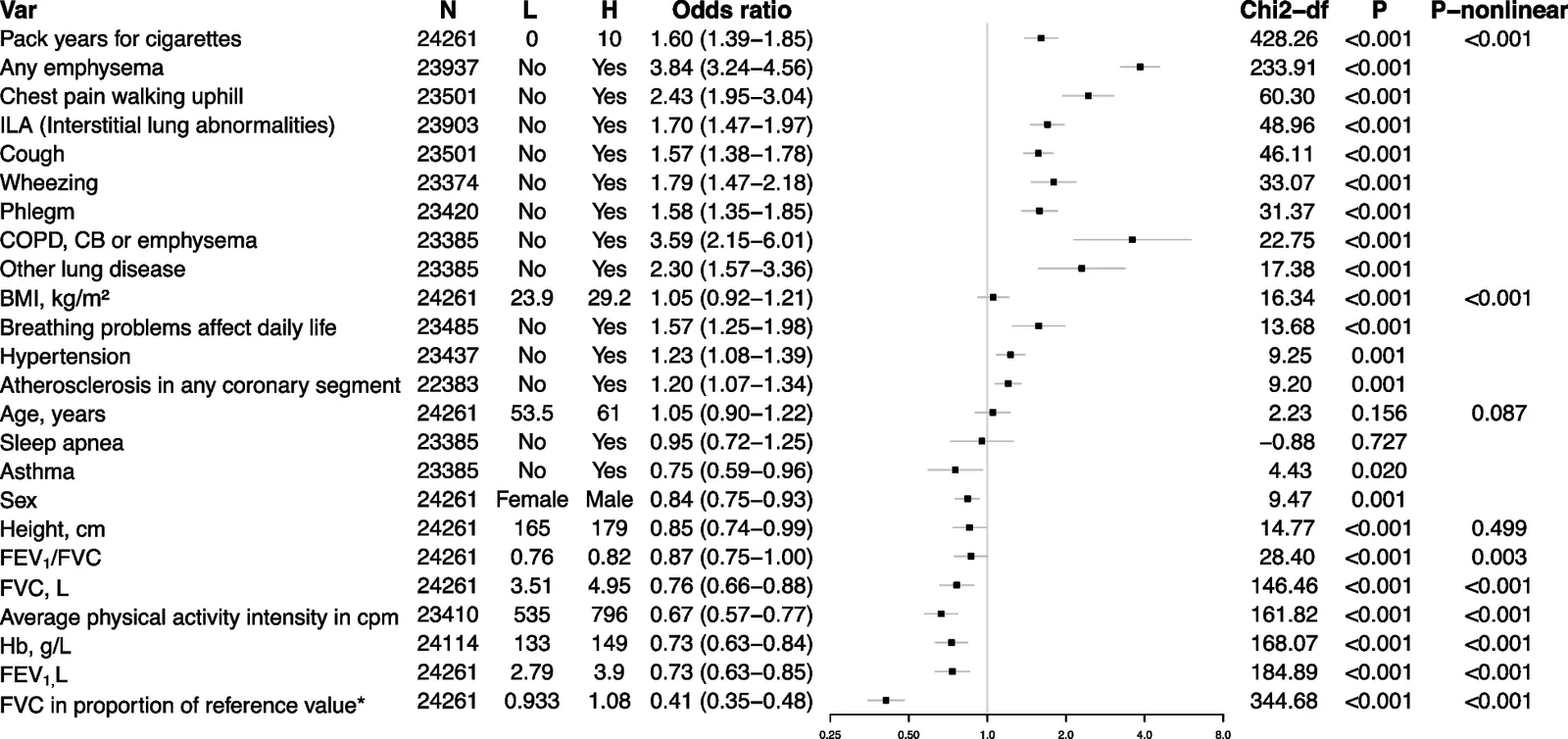
Forest plot showing odds ratios for univariable analysis of main variables. Odds ratios for the continuous variables are for the change between the 25th (L) and 75th (H) percentile while the odds ratios for categorical values are between no and yes. *Indicates SCAPIS reference equations [14]

The two variables that were most strongly associated with increased risk of D_LCO_ < LLN, based on significance (*x*^*2*^), were smoking history and emphysema (Fig. 2). In contrast, higher levels of FVC, Hb, physical activity and FEV_1_ were most strongly associated with reduced risk of D_LCO_ < LLN. Plots of relative ORs from the univariable analyses of the main variables can be found in Supplementary Fig. 1.

**Fig. 3:**
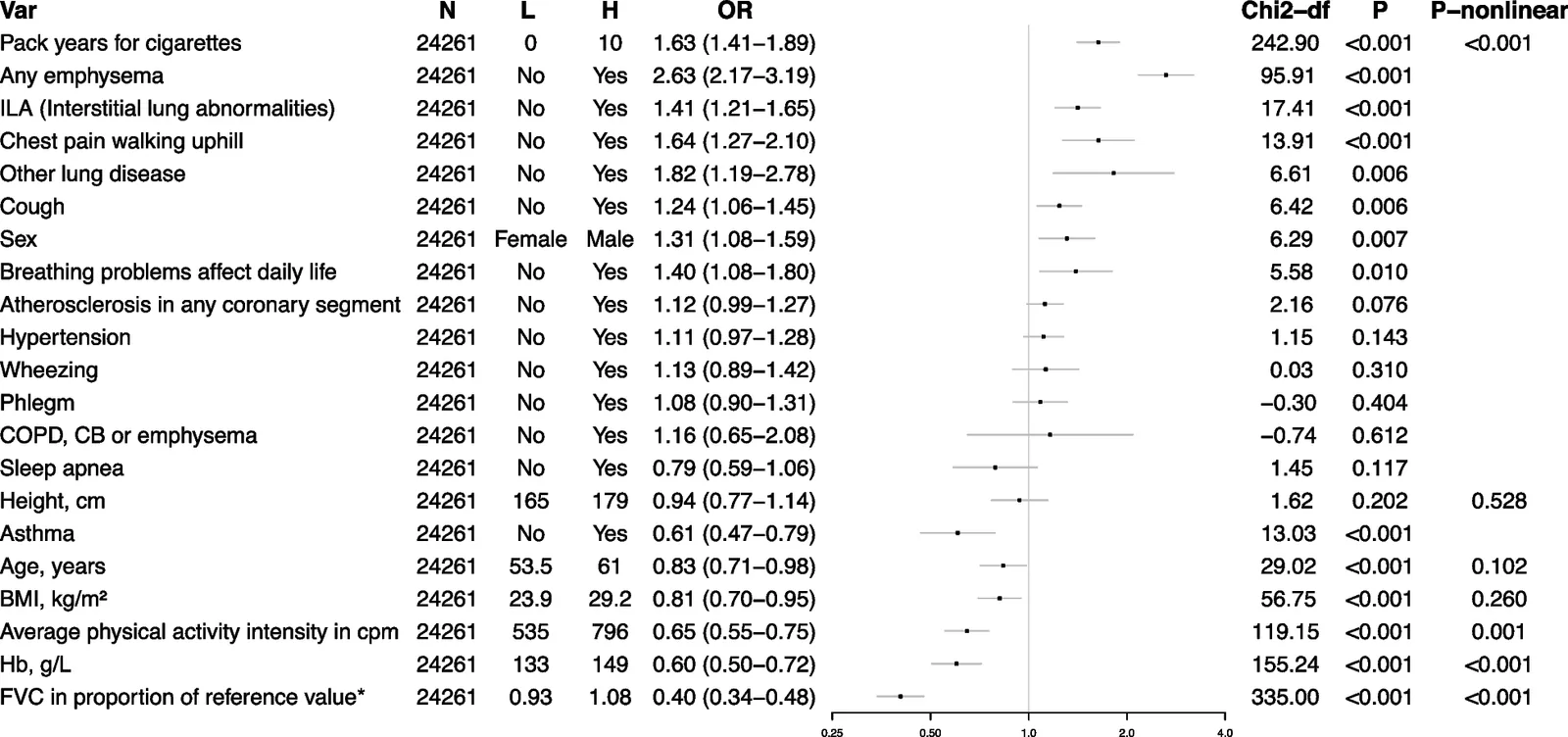
Forest plot showing odds ratios for multivariable analysis of main variables. Odds ratios for the continuous variables are for the change between the 25th (L) and 75th (H) percentile while the odds ratios for categorical values are between no and yes. *Indicates SCAPIS reference equations [14]

In the multivariable analysis of the main variables, smoking history, emphysema, ILA, other lung disease and reported symptoms of chest pain when walking uphill showed the strongest associations based on significance (*x*^*2*^). The effect size was largest for emphysema (Fig. 3). The results were essentially unchanged after adding the Z-score for VA to the model (Supplementary Fig. 2). Plots of relative ORs for multivariable analysis of the main variables can be found in Supplementary Fig. 3.

There was a significant association between reported chest pain when walking uphill and prevalence of atherosclerosis in any coronary segment (*P* = 0.008). However, atherosclerosis did not increase the risk of D_LCO_ < LLN (OR 1.12, 95% confidence interval (CI) 0.99–1.27) and there was no interaction between chest pain and atherosclerosis on the risk of having reduced D_LCO_ (P_interaction_ = 0.86). Heart failure and diabetes mellitus did not associate with the risk of D_LCO_ < LLN (data not shown).

The main factors related to D_LCO_ < LLN were confirmed in a complete case analysis (Supplementary Fig. 4). Furthermore, a multivariable model without age as a covariate yielded similar results as the multivariable model with age included (Supplementary Fig. 5).

### Interactions with smoking status

**Fig. 4:**
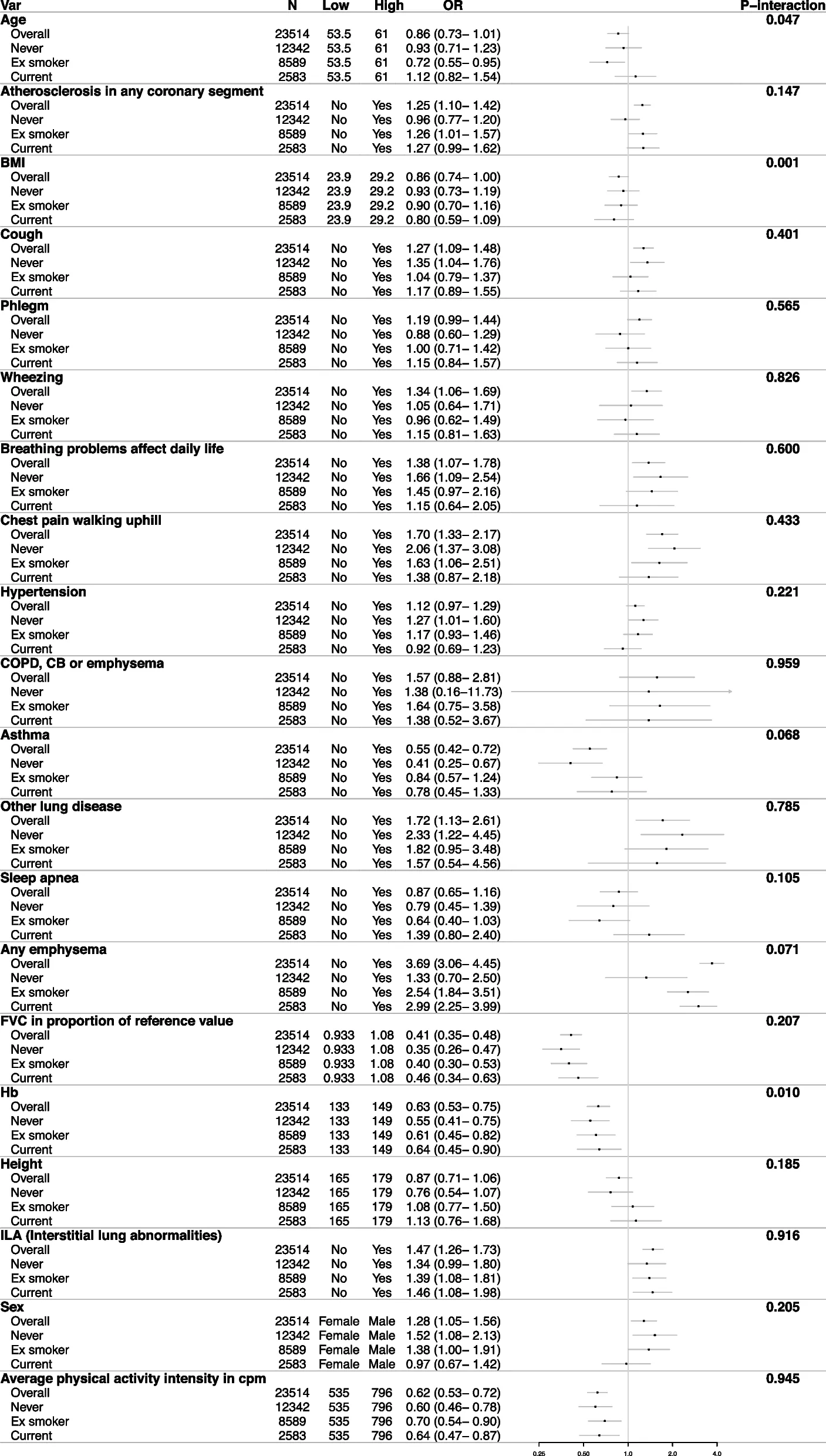
Forest plot showing odds ratios for multivariable analysis of main variables (Pack years for cigarettes excluded), stratified by smoking status. Odds ratios for the continuous variables are shows for the change between the 25th (L) and 75th (H) percentile while the odds ratios for categorical values are shown between no and yes

Three variables showed significant interaction with smoking status: age, Hb and BMI. The latter showed the most obvious interaction, with a gradually lower OR from never smokers to ex-smokers and on to current smokers (Fig. 4).

### Overlap and interactions between ILA, emphysema and reduced DLCO

A total of 206 subjects (4%) had both radiological ILA and emphysema. The combination of ILA and emphysema on CT was associated with the highest risk of having D_LCO_ < LLN (OR 3.74, 95% CI 2.94–4.75). However, there was no interaction between ILA and emphysema on the risk of having reduced D_LCO_ (P_interaction_ = 0.701). The overlap between ILA, emphysema and D_LCO_ < LLN in the study population is shown in Supplementary Fig. 6.

## Discussion

Here, we report determinants of having isolated, reduced D_LCO_ in a large population-based sample of middle-aged subjects. The strongest association was found with emphysema on CT, but associations were also observed with ILA on CT and self-reported lung disease, other than asthma or COPD, that had been diagnosed by a physician. More unexpectedly and of particular interest, the self-reported symptom of chest pain when walking uphill was strongly associated with isolated, reduced D_LCO_. A lower likelihood of having isolated, reduced D_LCO_ was found with asthma, higher BMI, higher age, higher physical activity, higher Hb and/or higher FVC.

### DLCO in relation to emphysema

The finding of an association between reduced D_LCO_ and emphysema even with normal spirometry has previously been assessed in a number of studies. Klein [7] reviewed examinations of emphysema through CT among smokers and could establish a correlation between D_LCO_ and a visual score of emphysema in a small number of subjects with abnormal D_LCO_ but without airflow obstruction. Aduen et al*.* [8] reported that emphysema, frequently in combination with interstitial lung disease, was present in approximately half of the subjects with reduced D_LCO_ but normal spirometry and static lung volumes. Interstitial lung disease alone was present in approximately one fifth of the subjects. In a prospective study, Kirby et al*.* [9] compared ex-smokers with normal spirometry and normal or reduced D_LCO_ using magnetic resonance imaging and hyperpolarised He-3. Subjects with reduced D_LCO_ had a higher apparent diffusion coefficient, indicating mild emphysema. They also had shorter six-minute walking distance and more symptoms, as assessed with the St George’s respiratory questionnaire, than their counterparts with normal D_LCO_. No difference could be detected between the groups through CT. A longitudinal study of smokers with normal spirometry and normal or reduced D_LCO_ was performed by Harvey et al*.* [10]. At inclusion, subjects with reduced D_LCO_, at the group level, had lower mean slow vital capacity, FEV_1_, FEV_1_/FVC and total lung capacity. The groups did not differ in smoking habits, symptoms of coughing or dyspnoea, or in the extent of emphysema detected by CT. During the follow-up period, 22% of subjects with low D_LCO_ developed airflow limitation compared with only 3% in the group with normal D_LCO_ at inclusion. In the latter group, D_LCO_ fell below the LLN during the follow-up period, whereas spirometry remained essentially unchanged. Taken together, the previous studies indicate that an isolated, reduced D_LCO_ reflects mild emphysema, possibly in combination with other pathology and that subjects with this finding have an increased risk of developing COPD. Pursuant to the expanded diagnostic criteria for COPD suggested by the Lancet commission [20], the finding of isolated, reduced D_LCO_ in a symptomatic subject might even be regarded as diagnostic, if there is clinical suspicion and other reasons have been excluded.

### DLCO in relation to ILA and lung disease other than COPD or asthma

Aside from emphysema, ILA with fibrosis is also a known cause of reduced D_LCO,_ which was an expected finding in our current study. Reduced D_LCO_ is a more sensitive marker of fibrosis than spirometry. In a recent study, reduced D_LCO_ was the only significant predictor of mortality in progressive pulmonary fibrosis [21]. It is well-known that smokers are more likely to show decreasing D_LCO_ with advancing age, especially in the case of pulmonary fibrosis [22]. Recent Mendelian studies have indicated that smoking indeed seems to be causally related to decreasing D_LCO_ in such patients [23].

### Factors associated with a lower likelihood of reduced DLCO

Factors associated with a lower likelihood of having isolated reduced D_LCO_ were asthma, higher BMI, higher age, higher physical activity, higher Hb and higher FVC. Asthma is not expected to reduce D_LCO_ [24] and may even be related to higher D_LCO_ [25], in line with our current findings. Moreover, higher FVC makes impaired D_LCO_ less likely [5]. Higher Hb is also expected to be associated with higher D_LCO_, reflected by the fact that anaemia is one of the factors associated with impaired D_LCO_ and corrected for in clinical practice [5]. Higher physical activity may reflect that study participants with normal lung function were able to exercise more than subjects with impaired D_LCO_, as they would have normal gas exchange during exercise. These findings are in line with findings from a recent study showing that D_LCO_ is associated with VO₂ max, regardless of severity of airflow limitation and emphysema [26]. Higher BMI was related to increased D_LCO_ in a previous study [25], although a more recent study did not find any relation between BMI > 30 kg/m^2^ and D_LCO_ in a population with a similar prevalence of BMI ≥ 30 kg/m^2^ as that seen in the present material [27]. The association between higher age and lower likelihood of isolated, reduced D_LCO_ is hard to interpret, as age is part of the equation for assessing the LLN of D_LCO_ in SCAPIS. Also, the age range in SCAPIS is narrow.

### Extrapulmonary factors and symptoms associated with DLCO

The finding that reporting symptoms of chest pain when walking uphill was strongly associated with isolated, reduced D_LCO_ merits some special consideration. Whether or not chest pain is a common consequence of, or cause of, reduced D_LCO_ cannot be determined based on the current study and the potential causes can only be speculated on. Potential common mechanisms could involve damage to elastin in both arteries and alveoli [28–31] or endothelial dysfunction in both the coronary and pulmonary vasculature [32]. Another factor could be related to West’s observation that increased pulmonary capillary pressure triggers rapid remodelling of the alveolar-capillary membrane, as increased membrane thickness is required to withstand the elevated pressure [33]. Another possible explanation is that subclinical restrictive lung disease may contribute to both chest discomfort and reduced D_LCO_. However, participants with spirometric suspicion of restriction (low FVC) were excluded from the present analyses and the observed association persisted after adjustment for FVC. Nevertheless, since static lung volumes were not available, this possibility cannot be completely excluded. A previous population-based study found a link between carotid plaques and low D_LCO_, but not FEV_1_ or COPD status, even after adjusting for C-reactive protein and traditional atherosclerosis risk factors [34]. We have previously shown that coronary artery disease (CAD) seems to be related to reduced D_LCO_ in high-risk patients who were examined due to suspected CAD. The results were unchanged even after accounting for lung function and heart failure in these patients [35]. Chest pain when walking uphill may be a symptom of CAD and it would be tempting to speculate that reduced D_LCO_ may be an early marker of CAD in a population-based setting. However, in the current study, coronary atherosclerosis did not associate with reduced D_LCO_ and there was no significant interaction between chest pain and coronary atherosclerosis on the risk of having reduced D_LCO_. Thus, the association between chest pain during exertion and reduced D_LCO_ was not explained by concurrent CAD in our study population. It should be emphasized that chest pain was assessed using a self-reported questionnaire item and was not verified through clinical evaluation. Consequently, symptom misclassification is possible, and the reported symptom may reflect a heterogeneous group of underlying conditions rather than clinically confirmed angina. Therefore, this finding should be regarded as hypothesis-generating and warrants confirmation in future studies.

Heart failure [2] and diabetes [36] are conditions that are known to be associated with reduced D_LCO_. In a retrospective study of patients with a diagnosis of pulmonary hypertension due to heart failure with preserved ejection fraction, lower D_LCO_ was strongly associated with higher mortality [37]. However, in the current study, we did not find any associations between isolated, reduced D_LCO_ and these conditions. Notably, the prevalence of heart failure in particular, but also of dysregulated diabetes, was low in our cohort of generally healthy middle-aged subjects (Supplementary Table 2), which may explain these findings.

### Study strengths and limitations

The strengths of the present study are the large study population with information on imaging available for both lungs and coronary arteries, as well as information on comorbidities. The large study population enabled investigation of possible interactions with smoking habits. A limitation of the study is the narrow age range. Ethnicity was not registered and therefore not studied as a potential determinant of isolated reduced D_LCO_. This may limit the generalizability of our findings to populations with different demographic compositions. Participation in SCAPIS was voluntary and approximately half of invited individuals agreed to participate. Consequently, some degree of selection bias cannot be excluded. Previous analyses of participation in SCAPIS have shown that participation was mainly associated with sociodemographic characteristics, while disease history contributed less to participation patterns [38]. Furthermore, an evaluation of selection effects in the full SCAPIS cohort suggested that the impact on the distribution of cardiopulmonary risk factors at baseline was generally small [39]. Nevertheless, prevalence estimates of smoking history, symptoms and respiratory abnormalities should be interpreted with caution, as they may not fully reflect those of the underlying source population. In the current study, haemoglobin was included as a separate covariate in the analyses, allowing us to assess its independent contribution to the likelihood of having a reduced D_LCO_. Although haemoglobin-corrected D_LCO_ is commonly used in clinical practice, clinically significant anaemia was uncommon in our cohort and is therefore unlikely to explain the main findings. It should be noted that the meaning of “isolated, reduced D_LCO_” in our study means reduced D_LCO_ with normal spirometry, i.e. static lung volumes were not measured. Furthermore, the fact that diseases that had been diagnosed by a physician were self-reported can also be perceived as a limitation, although this is common and feasible in epidemiological studies. Lastly, the fact that we had a relatively large proportion (almost 20%) of subjects lacking information on one or more of the variables of interest is also a limitation. However, we addressed this through multiple imputations and could confirm the main findings through complete case analyses.

## Conclusion

In conclusion, we present factors associated with isolated, reduced diffusing capacity from a large population-based study. The associations with emphysema and smoking history should guide clinicians to consider pre-COPD where there is clinical suspicion. Furthermore, the association with interstitial lung changes and emphysema suggests that there is a complementary value in performing CT of the lungs in subjects with isolated reduction of D_LCO_. The association between isolated, reduced D_LCO_ and reported chest pain during exertion is interesting and warrants further study. The present findings should be confirmed in further studies and with other age ranges.

## Supplementary Information


Supplementary Material 1.
Supplementary Material 2.
Supplementary Material 3.


## Data Availability

The datasets generated and/or analysed during the current study are not publicly available due to legal requirements but anonymized datasets are available from the corresponding author on reasonable request.
