# Supplementary material for: Isolated, reduced diffusing capacity for carbon monoxide in the population – relations to symptoms, smoking and comorbidities
Source: BMC Pulm Med. 2026 Aug 31;26:387. doi: 10.1186/s12890-026-04662-9 (PMC13531966; doi:10.1186/s12890-026-04662-9)
Supplement: Supplementary file 1 [file 12890_2026_4662_MOESM2_ESM.pdf]

Supplementary Table 1. Descriptive Statistics

	N	Normal spirometry and DLco $N = 22789$	Normal spirometry; DLco<LLN $N = 1472$
**Basic**			
Age	28855	57.2 (53.5 – 61.0)	57.2 (53.4 – 61.2)
Sex: Female	28855	11796 (51.8%)	826 (56.1%)
Male		10993 (48.2%)	646 (43.9%)
**Anthropometry**			
BMI, kg/m^2	28855	26.3 (23.9 – 29.2)	26.1 (23.6 – 29.1)
Height, cm	28855	172.0 (165.0 – 179.0)	171.0 (164.8 – 178.0) 171.0
Any emphysema	28435	792 (3.5%) [299]	178 (12.3%) [25]
Ground glass opacity found on CT	28397	1321 (5.9%) [321]	123 (8.5%) [27]
Honeycombing found on CT	28433	31 (0.1%) [299]	6 (0.4%) [25]
Reticular abnormality found on CT	28440	262 (1.2%) [295]	39 (2.7%) [26]
Cysts	28433	487 (2.2%) [301]	60 (4.1%) [24]
Bronchis	28434	500 (2.2%) [299]	77 (5.3%) [24]
ILA (Interstitial lung abnormalities)	28385	2290 (10.2%) [328]	233 (16.2%) [30]
Coronary artery calcium score according to Agatston	27832	0.0 (0.0-16.0) [709]	0.0 (0.0-29.0) [71]
Atherosclerosis in any coronary segment	26429	8714 (41.3%) [1702]	594 (45.8%) [176]
Presence of non-calcified plaques in the coronary segments	25773	1242 (6.0 %) [2226]	105 (8.3 %) [212]
Presence of any carotid artery plaque:			
One vessel		6843 (30.0%)	397 (27.0%)
Both vessels		5290 (23.2%)	430 (29.2%)
Not applicable		101 (0.4%)	13 (0.9%)
**Biochemistry**			
eGFR CKD-Epi	28796	89.6 (80.2 – 98.7) [42]	89.4 (78.9 – 99.1) [4]
Rev Malmo-Lund	28796	76.8 (70.8 – 83.1) [42]	77.0 (70.1 – 83.6) [4]
Creatinine	28796	77.0 (68.0 – 87.0) [42]	75.0 (67.0 – 87.0) [4]
Glucose	26404	5.6 (5.2 – 6.0) [1878]	5.6 (5.2 – 6.0) [150]
Hba1c	28707	35.0 (33.0 – 38.0) [104]	36.0 (34.0 – 39.0) [10]
Hb	28665	141.0 (133.0 – 150.0) [137]	138.0 (130.0 – 147.0) [10]
Total cholestrerol	28776	5.5 (4.8 – 6.2) [58]	5.4 (4.7 – 6.2) [4]
HDL	28770	1.6 (1.3 – 1.9) [62]	1.5 (1.2 – 1.8) [4]
LDL	28636	3.4 (2.8 – 4.0) [157]	3.4 (2.8 – 4.2) [7]
TG	28776	1.0 (0.8 – 1.4) [60]	1.2 (0.9 – 1.7) [3]
CRP	28777	0.9 (0.6 – 2.0) [56]	1.4 (0.7 – 2.9) [3]
**Lung function**			
Chronic airflow limitation: Having	28855	0 (0.0%)	0 (0.0%)
DLco, mmol min-1 kPa-1	28855	8.6 (7.3 – 10.1)	6.1 (5.5 – 7.2)
K_{CO}	28851	1.4 (1.3 – 1.6) [3]	1.2 (1.1 – 1.3) [0]
FEV1, L	28855	3.3 (2.8 – 3.9)	3.0 (2.6 – 3.6)
FEV1/VCmax	28855	0.78 (0.75 – 0.81)	0.77 (0.74 – 0.81)
FEV1/FVC	28855	0.79 (0.76 – 0.82)	0.79 (0.75 – 0.82)
FVC, L	28855	4.2 (3.5 – 5.0)	3.9 (3.3 – 4.6)
MEF50	28851	3.9 (3.2 – 4.8) [1]	3.7 (3.0 – 4.5) [0]
SVC	28723	4.2 (3.5 – 5.0) [104]	3.9 (3.3 – 4.6) [4]
**Blood pressure**			
Systolic blood pressure	28791	124.0 (114.0 – 136.0) [40]	120.0 (110.0 – 132.2) [4]
Diastolic blood pressure	28789	77.0 (70.0 – 84.0) [40]	74.0 (68.0 – 82.0) [4]
The ankle-brachial index left	28686	1.2 (1.2 – 1.3) [122]	1.2 (1.2 – 1.3) [11]
The ankle-brachial index right	28675	1.2 (1.2 – 1.3) [125]	1.2 (1.2 – 1.3) [14]
**Physical activity**			
Average physical activity intensity in cpm	27825	662.2 (538.2 – 800.5) [780]	601.8 (480.8 – 729.8) [71]

m $(a - b)$ represents median (Q_1 – Q_3).
n (p%) represent frequency (percentage). Percentages computed by group.
[M] represents number of missings.

Supplementary Table 1 Continued

	N	Normal spirometry and DLco $N = 22789$	Normal spirometry; DLco<LLN $N = 1472$
**Health history**			
Myocardial infarction	27786	263 (1.2%) [795]	37 (2.7%) [81]
Atrial Fibrillation	27786	348 (1.6%) [795]	27 (1.9%) [81]
Heart failure	27786	78 (0.4%) [795]	21 (1.5%) [81]
Heart valve disease	27786	155 (0.7%) [795]	20 (1.4%) [81]
CABG or PCI	27786	174 (0.8%) [795]	25 (1.8%) [81]
PAD, intervention	27786	52 (0.2%) [795]	3 (0.2%) [81]
Stroke	27786	268 (1.2%) [795]	36 (2.6%) [81]
Hypertension	27855	4734 (21.5%) [753]	352 (25.1%) [71]
Hyperlipidemia	27855	2440 (11.1%) [753]	210 (15.0%) [71]
Diabetes	27855	825 (3.7%) [753]	79 (5.6%) [71]
Glycaemic status: Normoglycemia	28828	17864 (78.5%) [20]	1068 (72.6%) [1]
Known diabetes		908 (4.0%)	86 (5.8%)
New diabetes		566 (2.5%)	51 (3.5%)
IFG		2949 (13.0%)	211 (14.3%)
Elevated HBA1C		482 (2.1%)	55 (3.7%)
COPD, CB or emphysema	27786	80 (0.4%) [795]	18 (1.3%) [81]
COPD	313	39 (55.7%) [22719]	10 (62.5%) [1456]
CB	298	30 (42.9%) [22719]	5 (31.2%) [1456]
Emphysema	288	7 (10.4%) [22722]	5 (33.3%) [1457]
COPD, CB or emphysema medication last two weeks	27781	28 (0.1%) [796]	9 (0.6%) [81]
Asthma	27786	1547 (7.0%) [795]	75 (5.4%) [81]
Asthma medication last two weeks	27774	814 (3.7%) [804]	42 (3.0%) [81]
Other lung disease	27786	216 (1.0%) [795]	31 (2.2%) [81]
Sleep apnea	27786	928 (4.2%) [795]	56 (4.0%) [81]
Sleep apnea treatment	27769	642 (2.9%) [808]	38 (2.7%) [81]
**Symptoms**			
Cough	27913	3652 (16.5%) [686]	331 (23.7%) [74]
Cough for three months	5063	1720 (48.6%) [19249]	160 (50.5%) [1155]
Phlegm	27805	2050 (9.3%) [765]	195 (14.0%) [76]
Phlegm for three months	2982	1135 (57.6%) [20818]	104 (55.0%) [1283]
Wheezing	27748	1112 (5.1%) [803]	121 (8.7%) [84]
Wheezing last 12 months	2007	979 (88.9%) [21688]	111 (91.7%) [1351]
Wheezing when cold, last 12 months	1941	400 (37.5%) [21722]	44 (36.7%) [1352]
Wheezing and dyspnea	1968	276 (25.4%) [21701]	33 (27.7%) [1353]
Dyspnea wheen hurrying	27830	1589 (7.2%) [740]	222 (15.9%) [77]
Dyspnea wheen walking plane	2421	607 (43.3%) [21386]	90 (45.2%) [1273]
Need to stop when walking	2651	188 (12.1%) [21229]	37 (16.9%) [1253]
Need to stop after 100 meters	2652	84 (5.4%) [21229]	24 (10.9%) [1252]
Breatlessness prevent leaving home	2658	73 (4.7%) [21226]	15 (6.8%) [1253]
Breathing problems affect daily life	27888	866 (3.9%) [699]	84 (6.0%) [77]
Number of episodes with breating problems last 12 months	1226	0.0 (0.0 – 1.0) [21975]	1.0 (0.0 – 2.0) [1394]
Number of episodes with breating problems last 12 months requiring health care	1205	0.0 (0.0 – 1.0) [21989]	0.0 (0.0 – 1.0) [1397]
Number of episodes with breating problems last 12 months requiring hospital care	1185	0.0 (0.0 – 0.0) [21998]	0.0 (0.0 – 0.0) [1400]
Number of epsiodes with airway problems requiring hospital care last 12 months	9886	591 (7.5%) [14884]	43 (8.6%) [973]
Chest pain walking uphill	27914	641 (2.9%) [690]	95 (6.8%) [70]
Chest pain walking on plain ground	27982	153 (0.7%) [630]	32 (2.3%) [73]
Breathlessness when climing two stairs	982	367 (57.0%) [22145]	62 (68.1%) [1381]
Hours of sleep per night	27740	3.0 (2.0 – 3.0) [824]	3.0 (2.0 – 3.0) [88]
Frequency of difficulty falling asleep	27775	1.0 (0.0 – 2.0) [793]	1.0 (0.0 – 2.0) [85]
Frequency of waking up	27740	2.0 (1.0 – 3.0) [816]	2.0 (1.0 – 4.0) [88]
Frequency of waking early	27711	1.0 (0.0 – 2.0) [844]	1.0 (0.0 – 2.0) [90]

m $(a - b)$ represents median $(Q_1 - Q_3)$.
n $(p\%)$ represent frequency (percentage). Percentages computed by group.
$[M]$ represents number of missings.

Supplementary Table 1 Continued. Descriptive statistics

	N	Normal spirometry and DLco $N = 22789$	Normal spirometry; DLco<LLN $N = 1472$
**Smoking**			
Smoking status: Never	28002	11841 (53.6%) [705]	501 (35.0%) [42]
Ex smoker		8093 (36.6%)	496 (34.7%)
Current		2150 (9.7%)	433 (30.3%)
Smoking duration in years	28855	0.0 (0.0 – 19.0)	17.0 (0.0 – 37.0)
Pack years for cigarettes	28855	0.0 (0.0 – 9.5)	7.5 (0.0 – 22.0)

m (a – b) represents median (Q_1 – Q_3).
n (p%) represent frequency (percentage). Percentages computed by group.
[M] represents number of missings.

Supplementary Table 2. Descriptive Statistics, participants with normal spirometry split by smoking.

	Never smoked			Ex smoker			Current smoker		
	N	Normal DLco $N = 11841$	DLco<LLN $N = 501$	N	Normal DLco $N = 8093$	DLco<LLN $N = 496$	N	Normal DLco $N = 2150$	DLco<LLN $N = 433$
**Basic**									
Age	12342	56.5 (53.1 – 60.5)	56.5 (52.7 – 61.1)	8589	58.5 (54.6 – 61.9)	57.8 (53.9 – 61.5)	2583	56.5 (53.2 – 60.2)	57.5 (53.5 – 60.8)
Sex: Female	12342	5813 (49.1%)	286 (57.1%)	8589	4528 (55.9%)	280 (56.5%)	2583	1113 (51.8%)	243 (56.1%)
Male		6028 (50.9%)	215 (42.9%)		3565 (44.1%)	216 (43.5%)		1037 (48.2%)	190 (43.9%)
**Anthropometry**									
BMI, kg/m²	12342	26.0 (23.6 – 28.8)	26.1 (23.6 – 29.2)	8589	26.6 (24.2 – 29.6)	26.4 (23.7 – 29.5)	2583	26.5 (24.2 – 29.5)	25.9 (23.5 – 28.4)
Height, cm	12342	173.0 (166.0 – 180.0)	171.0 (164.0 – 178.0)	8589	171.0 (165.0 – 178.0)	171.0 (165.0 – 178.0)	2583	171.0 (165.0 – 179.0)	170.0 (164.0 – 177.0)
**Imaging**									
Any emphysema	12196	213 (1.8%) [140]	11 (2.2%) [6]	8475	356 (4.5%) [109]	53 (10.8%) [5]	2543	208 (9.8%) [26]	109 (26.0%) [14]
Ground glass opacity found on CT	12181	606 (5.2%) [154]	35 (7.1%) [7]	8471	547 (6.9%) [114]	42 (8.5%) [4]	2538	132 (6.2%) [29]	41 (9.8%) [16]
Honeycombing found on CT	12196	16 (0.1%) [140]	0 (0.0%) [6]	8477	11 (0.1%) [108]	1 (0.2%) [4]	2541	4 (0.2%) [27]	4 (1.0%) [15]
Reticular abnormality found on CT	12196	129 (1.1%) [138]	6 (1.2%) [8]	8478	97 (1.2%) [107]	17 (3.5%) [4]	2543	30 (1.4%) [26]	13 (3.1%) [14]
Cysts	12193	196 (1.7%) [143]	13 (2.6%) [6]	8478	202 (2.5%) [107]	19 (3.9%) [4]	2542	76 (3.6%) [27]	25 (6.0%) [14]
Bronchis	12197	220 (1.9%) [139]	15 (3.0%) [6]	8478	206 (2.6%) [107]	34 (6.9%) [4]	2540	65 (3.1%) [29]	24 (5.7%) [14]
ILA (Interstitial lung abnormalities)	12174	1040 (8.9%) [159]	56 (11.4%) [9]	8469	934 (11.7%) [116]	82 (16.7%) [4]	2537	258 (12.2%) [29]	86 (20.7%) [17]
Coronary artery calcium score according to Agatston	11992	0.0 (0.0 – 9.0) [324]	0.0 (0.0 – 8.5) [26]	8278	0.0 (0.0 – 25.0) [288]	0.0 (0.0 – 32.0) [23]	2503	0.0 (0.0 – 36.0) [59]	1.5 (0.0 – 57.5) [21]
Atherosclerosis in any coronary segment	11462	4106 (37.3%) [825]	149 (33.4%) [55]	7889	3359 (45.1%) [642]	219 (50.0%) [58]	2365	965 (48.5%) [160]	207 (55.2%) [58]
Presence of non-calcified plaques in the coronary segments	11172	548 (5.1%) [1108]	23 (5.2%) [62]	7707	493 (6.8%) [813]	36 (8.4%) [69]	2298	161 (8.3%) [212]	44 (12.2%) [73]
Presence of any carotid artery plaque: No plaque	12342	5869 (49.6%)	268 (53.5%)	8589	3523 (43.5%)	202 (40.7%)	2583	853 (39.7%)	143 (33.0%)
One vessel		3469 (29.3%)	139 (27.7%)		2473 (30.6%)	139 (28.0%)		671 (31.2%)	111 (25.6%)
Both vessels		2468 (20.8%)	92 (18.4%)		2056 (25.4%)	150 (30.2%)		606 (28.2%)	173 (40.0%)
Not applicable		35 (0.3%)	2 (0.4%)		41 (0.5%)	5 (1.0%)		20 (0.9%)	6 (1.4%)
**Biochemistry**									
eGFR CKD-Epi	12321	88.6 (79.7 – 98.0) [20]	86.4 (76.8 – 96.2) [1]	8572	89.7 (80.1 – 98.6) [16]	87.2 (77.4 – 98.3) [1]	2579	94.9 (84.0 – 101.6) [2]	95.9 (84.6 – 101.6) [2]
eGFR revised Malmö-Lund	12321	76.3 (70.4 – 82.4) [20]	75.4 (68.5 – 81.2) [1]	8572	76.7 (70.7 – 82.9) [16]	75.6 (69.1 – 83.2) [1]	2579	80.2 (73.7 – 86.3) [2]	80.2 (74.1 – 86.2) [2]
Creatinine	12321	78.0 (69.0 – 88.0) [20]	77.0 (69.0 – 89.0) [1]	8572	76.0 (67.0 – 85.0) [16]	76.0 (67.0 – 88.0) [1]	2579	74.0 (64.0 – 84.0) [2]	72.0 (64.0 – 81.0) [2]
Glucose	11486	5.5 (5.2 – 5.9) [813]	5.6 (5.2 – 6.0) [43]	7767	5.6 (5.2 – 6.0) [775]	5.6 (5.2 – 6.0) [47]	2281	5.6 (5.2 – 6.0) [245]	5.6 (5.2 – 6.0) [57]
Hba1c	12291	35.0 (33.0 – 38.0) [48]	36.0 (34.0 – 39.0) [3]	8552	36.0 (33.0 – 38.0) [35]	36.0 (34.0 – 39.0) [2]	2563	36.0 (34.0 – 38.0) [15]	37.0 (35.0 – 40.0) [5]
Hb	12272	142.0 (133.0 – 150.0) [68]	137.0 (129.0 – 146.0) [2]	8541	140.0 (133.0 – 148.0) [46]	137.0 (129.0 – 146.0) [2]	2561	142.0 (134.0 – 151.0) [16]	140.0 (131.0 – 149.0) [6]
Total cholestrerol	12312	5.4 (4.8 – 6.1) [28]	5.3 (4.7 – 6.0) [2]	8571	5.5 (4.8 – 6.2) [18]	5.4 (4.7 – 6.2) [0]	2574	5.5 (4.8 – 6.2) [7]	5.5 (4.8 – 6.3) [2]
HDL	12313	1.6 (1.3 – 1.9) [28]	1.5 (1.2 – 1.8) [1]	8568	1.6 (1.3 – 2.0) [21]	1.5 (1.2 – 1.9) [0]	2573	1.5 (1.2 – 1.8) [8]	1.4 (1.1 – 1.8) [2]
LDL	12275	3.4 (2.8 – 4.0) [65]	3.3 (2.7 – 4.0) [2]	8534	3.4 (2.8 – 4.1) [54]	3.4 (2.7 – 4.1) [1]	2550	3.5 (2.8 – 4.1) [31]	3.6 (2.9 – 4.3) [2]
TG	12314	1.0 (0.7 – 1.4) [27]	1.1 (0.8 – 1.6) [1]	8569	1.0 (0.8 – 1.5) [20]	1.2 (0.9 – 1.7) [0]	2573	1.2 (0.8 – 1.7) [8]	1.3 (1.0 – 1.8) [2]
CRP	12317	0.9 (0.6 – 1.8) [24]	1.3 (0.6 – 2.6) [1]	8570	1.0 (0.6 – 2.1) [19]	1.4 (0.7 – 2.8) [0]	2575	1.1 (0.6 – 2.3) [6]	1.6 (0.8 – 3.2) [2]
**Lung function**									
Chronic airflow limitation: Having	12342	0 (0.0%)	0 (0.0%)	8589	0 (0.0%)	0 (0.0%)	2583	0 (0.0%)	0 (0.0%)
DLco, mmol min-1 kPa-1	12342	8.8 (7.5 – 10.3)	6.1 (5.5 – 7.4)	8589	8.3 (7.2 – 9.8)	6.1 (5.5 – 7.2)	2583	8.2 (7.0 – 9.5)	5.9 (5.3 – 7.1)
Kco	12341	1.5 (1.4 – 1.6) [1]	1.2 (1.1 – 1.3) [0]	8588	1.4 (1.3 – 1.6) [1]	1.2 (1.1 – 1.3) [0]	2582	1.4 (1.3 – 1.5) [1]	1.1 (1.0 – 1.2) [0]
FEV1, L	12342	3.4 (2.8 – 4.0)	3.0 (2.6 – 3.6)	8589	3.2 (2.8 – 3.8)	3.0 (2.6 – 3.5)	2583	3.2 (2.8 – 3.8)	3.0 (2.6 – 3.5)
FEV1/VCmax	12342	0.79 (0.75 – 0.82)	0.79 (0.76 – 0.82)	8589	0.78 (0.74 – 0.81)	0.77 (0.74 – 0.81)	2583	0.77 (0.74 – 0.81)	0.76 (0.72 – 0.79)
FEV1/FVC	12342	0.80 (0.76 – 0.83)	0.80 (0.77 – 0.84)	8589	0.79 (0.76 – 0.82)	0.79 (0.75 – 0.82)	2583	0.79 (0.75 – 0.82)	0.77 (0.73 – 0.80)
FVC, L	12342	4.3 (3.6 – 5.1)	3.9 (3.3 – 4.6)	8589	4.1 (3.5 – 4.8)	3.9 (3.3 – 4.5)	2583	4.1 (3.5 – 4.9)	3.9 (3.3 – 4.6)
MEF50	12341	4.0 (3.3 – 4.9) [1]	3.9 (3.2 – 4.7) [0]	8589	3.8 (3.1 – 4.7)	3.6 (3.0 – 4.4)	2583	3.8 (3.1 – 4.7)	3.4 (2.8 – 4.1)
SVC	12289	4.3 (3.6 – 5.1) [51]	3.8 (3.3 – 4.6) [2]	8546	4.1 (3.5 – 4.9) [43]	3.9 (3.3 – 4.6) [0]	2575	4.2 (3.5 – 5.0) [8]	4.0 (3.3 – 4.6) [0]
**Blood pressure**									
Systolic blood pressure	12331	124.0 (114.0 – 135.0) [11]	120.0 (111.0 – 132.0) [0]	8571	124.0 (114.0 – 136.0) [17]	122.0 (111.0 – 134.0) [1]	2573	122.0 (112.0 – 134.0) [7]	119.0 (108.0 – 132.0) [3]
Diastolic blood pressure	12331	77.0 (70.0 – 84.0) [11]	75.0 (68.0 – 82.0) [0]	8571	77.0 (70.0 – 84.0) [17]	76.0 (69.5 – 83.0) [1]	2573	76.0 (69.0 – 84.0) [7]	73.0 (68.0 – 82.0) [3]
The ankle-brachial index left	12301	1.2 (1.2 – 1.3) [40]	1.2 (1.2 – 1.3) [1]	8532	1.2 (1.2 – 1.3) [52]	1.2 (1.2 – 1.3) [5]	2564	1.2 (1.2 – 1.3) [14]	1.2 (1.1 – 1.3) [5]
The ankle-brachial index right	12298	1.2 (1.2 – 1.3) [43]	1.2 (1.2 – 1.3) [1]	8530	1.2 (1.2 – 1.3) [51]	1.2 (1.2 – 1.3) [8]	2564	1.2 (1.2 – 1.3) [14]	1.2 (1.1 – 1.3) [5]
**Physical activity**									
Average physical activity intensity in cpm	11940	667.9 (544.0 – 806.1) [377]	613.1 (485.7 – 730.2) [25]	8300	660.3 (535.1 – 796.1) [272]	612.5 (489.6 – 732.5) [17]	2467	642.7 (519.7 – 790.1) [90]	575.8 (461.4 – 724.0) [26]

m (a – b) represents median (Q_1 – Q_3).
n (p%) represent frequency (percentage). Percentages computed by group.
[M] represents number of missings.

Supplementary Table 2 Continued.

	Never smoked			Ex smoker			Current smoker		
	N	Normal DLco $N = 11841$	DLco<LLN $N = 501$	N	Normal DLco $N = 8093$	DLco<LLN $N = 496$	N	Normal DLco $N = 2150$	DLco<LLN $N = 433$
**Health history**									
Myocardial infarction: Yes	12198	112 (1.0%) [136]	15 (3.0%) [8]	8466	121 (1.5%) [114]	17 (3.5%) [9]	2428	29 (1.4%) [122]	5 (1.2%) [33]
Atrial Fibrillation: Yes	12198	179 (1.5%) [136]	8 (1.6%) [8]	8466	136 (1.7%) [114]	15 (3.1%) [9]	2428	27 (1.3%) [122]	4 (1.0%) [33]
Heart failure: Yes	12198	24 (0.2%) [136]	6 (1.2%) [8]	8466	45 (0.6%) [114]	11 (2.3%) [9]	2428	9 (0.4%) [122]	4 (1.0%) [33]
Heart valve disease: Yes	12198	75 (0.6%) [136]	7 (1.4%) [8]	8466	63 (0.8%) [114]	10 (2.1%) [9]	2428	16 (0.8%) [122]	3 (0.8%) [33]
CABG or PCI: Yes	12198	85 (0.7%) [136]	8 (1.6%) [8]	8466	78 (1.0%) [114]	11 (2.3%) [9]	2428	10 (0.5%) [122]	6 (1.5%) [33]
PAD, intervention: Yes	12198	24 (0.2%) [136]	0 (0.0%) [8]	8466	18 (0.2%) [114]	2 (0.4%) [9]	2428	9 (0.4%) [122]	1 (0.2%) [33]
Stroke: Yes	12198	127 (1.1%) [136]	14 (2.8%) [8]	8466	111 (1.4%) [114]	14 (2.9%) [9]	2428	26 (1.3%) [122]	8 (2.0%) [33]
Hypertension: Yes	12219	2265 (19.3%) [118]	123 (24.8%) [5]	8484	1960 (24.5%) [99]	136 (27.8%) [6]	2440	442 (21.7%) [113]	89 (22.1%) [30]
Hyperlipidemia: Yes	12219	1107 (9.4%) [118]	77 (15.5%) [5]	8484	1042 (13.0%) [99]	77 (15.7%) [6]	2440	262 (12.9%) [113]	54 (13.4%) [30]
Diabetes: Yes	12219	361 (3.1%) [118]	24 (4.8%) [5]	8484	357 (4.5%) [99]	31 (6.3%) [6]	2440	97 (4.8%) [113]	24 (6.0%) [30]
Glycaemic status: Normoglycemia	12332	9556 (80.8%) [10]	382 (76.2%) [0]	8582	6187 (76.5%) [6]	366 (73.9%) [1]	2582	1605 (74.7%) [1]	291 (67.2%) [0]
Known diabetes		383 (3.2%)	24 (4.8%)		379 (4.7%)	33 (6.7%)		105 (4.9%)	26 (6.0%)
New diabetes		265 (2.2%)	16 (3.2%)		208 (2.6%)	19 (3.8%)		59 (2.7%)	15 (3.5%)
IFG		1418 (12.0%)	66 (13.2%)		1132 (14.0%)	66 (13.3%)		307 (14.3%)	71 (16.4%)
Elevated HBA1C		209 (1.8%)	13 (2.6%)		181 (2.2%)	11 (2.2%)		73 (3.4%)	30 (6.9%)
COPD, CB or emphysema: Yes	12198	12 (0.1%) [136]	1 (0.2%) [8]	8466	52 (0.7%) [114]	9 (1.8%) [9]	2428	16 (0.8%) [122]	8 (2.0%) [33]
COPD: Yes	10	1 (11.1%) [11832]	0 (0.0%) [500]	56	28 (58.3%) [8045]	5 (62.5%) [488]	20	10 (76.9%) [2137]	5 (71.4%) [426]
CB: Yes	11	6 (60.0%) [11831]	1 (100.0%) [500]	58	18 (36.7%) [8044]	2 (22.2%) [487]	17	6 (54.5%) [2139]	2 (33.3%) [427]
Emphysema: Yes	10	2 (22.2%) [11832]	0 (0.0%) [500]	54	3 (6.4%) [8046]	3 (42.9%) [489]	18	2 (18.2%) [2139]	2 (28.6%) [426]
COPD, CB or emphysema medication last two weeks: Yes	12198	2 (0.0%) [136]	0 (0.0%) [8]	8466	21 (0.3%) [114]	5 (1.0%) [9]	2427	5 (0.2%) [123]	4 (1.0%) [33]
Asthma: Yes	12198	823 (7.0%) [136]	20 (4.1%) [8]	8466	599 (7.5%) [114]	34 (7.0%) [9]	2428	100 (4.9%) [122]	21 (5.2%) [33]
Asthma medication last two weeks: Yes	12194	424 (3.6%) [140]	12 (2.4%) [8]	8464	327 (4.1%) [116]	18 (3.7%) [9]	2425	51 (2.5%) [125]	12 (3.0%) [33]
Other lung disease: Yes	12198	101 (0.9%) [136]	12 (2.4%) [8]	8466	92 (1.2%) [114]	12 (2.5%) [9]	2428	17 (0.8%) [122]	7 (1.8%) [33]
Sleep apnea: Yes	12198	385 (3.3%) [136]	14 (2.8%) [8]	8466	448 (5.6%) [114]	21 (4.3%) [9]	2428	90 (4.4%) [122]	21 (5.2%) [33]
Sleep apnea treatment: Yes	12195	267 (2.3%) [139]	10 (2.0%) [8]	8460	315 (4.0%) [120]	14 (2.9%) [9]	2424	55 (2.7%) [126]	14 (3.5%) [33]
**Symptoms**									
Cough: Yes	12241	1704 (14.5%) [93]	97 (19.7%) [8]	8507	1341 (16.7%) [73]	89 (18.3%) [9]	2462	565 (27.5%) [96]	144 (35.3%) [25]
Cough for three months: Yes	1752	801 (48.3%) [10183]	41 (43.6%) [407]	1374	622 (48.2%) [6802]	45 (54.2%) [413]	690	279 (50.6%) [1599]	74 (53.2%) [294]
Phlegm: Yes	12196	885 (7.6%) [138]	41 (8.3%) [8]	8479	766 (9.6%) [100]	51 (10.5%) [10]	2450	379 (18.6%) [107]	103 (25.3%) [26]
Phlegm for three months: Yes	900	496 (57.7%) [10981]	17 (42.5%) [461]	781	416 (56.9%) [7362]	28 (56.0%) [446]	461	209 (57.7%) [1788]	59 (59.6%) [334]
Wheezing: Yes	12185	420 (3.6%) [147]	23 (4.7%) [10]	8465	424 (5.3%) [113]	30 (6.2%) [11]	2431	258 (12.7%) [120]	68 (17.0%) [32]
Wheezing last 12 months: Yes	437	354 (85.5%) [11427]	20 (87.0%) [478]	449	378 (90.2%) [7674]	27 (90.0%) [466]	326	238 (92.2%) [1892]	64 (94.1%) [365]
Wheezing when cold, last 12 months: Yes	426	162 (40.2%) [11438]	9 (39.1%) [478]	436	144 (35.4%) [7686]	13 (44.8%) [467]	315	90 (36.4%) [1903]	22 (32.4%) [365]
Wheezing and dyspnea: Yes	434	111 (27.0%) [11430]	6 (26.1%) [478]	444	113 (27.2%) [7678]	10 (34.5%) [467]	319	50 (19.8%) [1898]	17 (25.4%) [366]
Dyspnea wheen hurrying: Yes	12209	689 (5.9%) [124]	61 (12.4%) [9]	8496	669 (8.4%) [84]	71 (14.6%) [9]	2444	212 (10.4%) [111]	90 (22.2%) [28]
Dyspnea wheen walking plane: Yes	682	271 (43.2%) [11213]	26 (48.1%) [447]	643	253 (43.6%) [7513]	39 (61.9%) [433]	260	73 (41.0%) [1972]	25 (30.5%) [351]
Need to stop when walking: Yes	738	90 (13.3%) [11163]	6 (10.0%) [441]	725	62 (9.5%) [7438]	13 (18.6%) [426]	297	32 (15.4%) [1942]	18 (20.2%) [344]
Need to stop after 100 meters: Yes	736	37 (5.5%) [11166]	3 (4.9%) [440]	733	28 (4.2%) [7429]	13 (18.8%) [427]	293	18 (8.9%) [1947]	8 (8.9%) [343]
Breatlessness prevent leaving home: Yes	739	28 (4.1%) [11163]	2 (3.3%) [440]	726	25 (3.8%) [7436]	4 (5.8%) [427]	299	20 (9.5%) [1940]	9 (10.1%) [344]
Breathing problems affect daily life: Yes	12224	409 (3.5%) [114]	30 (6.0%) [4]	8513	376 (4.7%) [64]	35 (7.2%) [12]	2452	67 (3.3%) [101]	19 (4.7%) [30]
Number of episodes with breating problems last 12 months	419	0.0 (0.0 – 1.0) [11451]	0.0 (0.0 – 1.0) [472]	385	0.0 (0.0 – 1.0) [7740]	1.0 (0.0 – 2.2) [464]	75	0.0 (0.0 – 2.0) [2092]	2.0 (1.0 – 3.0) [416]
Number of episodes with breating problems last 12 months requiring health care	408	0.0 (0.0 – 1.0) [11460]	0.0 (0.0 – 1.0) [474]	381	0.0 (0.0 – 1.0) [7743]	1.0 (0.0 – 1.0) [465]	73	0.0 (0.0 – 1.0) [2094]	1.0 (0.0 – 2.0) [416]
Number of episodes with breating problems last 12 months requiring hospital care	402	0.0 (0.0 – 0.0) [11464]	0.0 (0.0 – 0.0) [476]	377	0.0 (0.0 – 0.0) [7747]	0.0 (0.0 – 0.0) [465]	71	0.0 (0.0 – 0.0) [2095]	0.0 (0.0 – 0.0) [417]
Number of epsiodes with airway problems requiring hospital care last 12 months: Yes	4695	306 (6.8%) [7341]	15 (7.7%) [306]	2860	231 (8.6%) [5404]	17 (9.9%) [325]	731	43 (7.1%) [1547]	11 (8.6%) [305]
Chest pain walking uphill: Yes	12234	283 (2.4%) [103]	35 (7.1%) [5]	8509	255 (3.2%) [72]	28 (5.7%) [8]	2462	98 (4.8%) [96]	32 (7.8%) [25]
Chest pain walking on plain ground: Yes	12267	73 (0.6%) [69]	9 (1.8%) [6]	8529	55 (0.7%) [51]	12 (2.5%) [9]	2466	25 (1.2%) [91]	11 (2.7%) [26]
Breathlessness when climing two stairs: Yes	318	156 (55.1%) [11558]	21 (60.0%) [466]	287	161 (61.7%) [7832]	19 (73.1%) [470]	125	47 (49.5%) [2055]	22 (73.3%) [403]
Hours of sleep per night	12168	3.0 (2.0 – 3.0) [160]	3.0 (2.0 – 3.0) [14]	8451	3.0 (2.0 – 3.0) [124]	3.0 (2.0 – 3.0) [14]	2437	3.0 (2.0 – 3.0) [118]	3.0 (2.0 – 3.0) [28]
Frequency of difficulty falling asleep	12184	1.0 (0.0 – 2.0) [146]	1.0 (0.0 – 2.0) [12]	8466	1.0 (0.0 – 2.0) [110]	1.0 (0.0 – 2.0) [13]	2438	1.0 (0.0 – 2.0) [118]	1.0 (0.0 – 3.0) [27]
Frequency of waking up	12174	2.0 (1.0 – 3.0) [155]	2.0 (1.0 – 4.0) [13]	8456	2.0 (1.0 – 4.0) [118]	2.0 (1.0 – 4.0) [15]	2432	2.0 (1.0 – 4.0) [122]	2.0 (1.0 – 4.0) [29]
Frequency of waking early	12161	1.0 (0.0 – 2.0) [167]	1.0 (0.0 – 2.0) [14]	8445	1.0 (0.0 – 2.0) [130]	1.0 (0.0 – 2.0) [14]	2425	1.0 (0.0 – 2.0) [127]	1.0 (0.0 – 2.0) [31]

m $(a - b)$ represents median $(Q_1 - Q_3)$.
n $(p\%)$ represent frequency (percentage). Percentages computed by group.
$[M]$ represents number of missings.

Supplementary Table 2 Continued.

	Never smoked			Ex smoker			Current smoker		
	N	Normal DLco $N = 11841$	DLco<LLN $N = 501$	N	Normal DLco $N = 8093$	DLco<LLN $N = 496$	N	Normal DLco $N = 2150$	DLco<LLN $N = 433$
**Smoking**									
Smoking status: Never	12342	11841 (100.0%)	501 (100.0%)	8589	0 (0.0%)	0 (0.0%)	2583	0 (0.0%)	0 (0.0%)
Ex smoker		0 (0.0%)	0 (0.0%)		8093 (100.0%)	496 (100.0%)		0 (0.0%)	0 (0.0%)
Current		0 (0.0%)	0 (0.0%)		0 (0.0%)	0 (0.0%)		2150 (100.0%)	433 (100.0%)
Smoking duration in years	12342	0.0 (0.0 – 0.0)	0.0 (0.0 – 0.0)	8589	18.0 (10.0 – 27.0)	22.0 (13.0 – 32.0)	2583	40.0 (36.0 – 44.0)	40.0 (37.0 – 45.0)
Pack years for cigarettes	12342	0.0 (0.0 – 0.0)	0.0 (0.0 – 0.0)	8589	9.5 (4.0 – 17.6)	12.9 (6.0 – 21.0)	2583	19.0 (10.0 – 30.0)	25.2 (16.0 – 33.8)

m $(a - b)$ represents median (Q_1 – Q_3).
n (p%) represent frequency (percentage). Percentages computed by group.

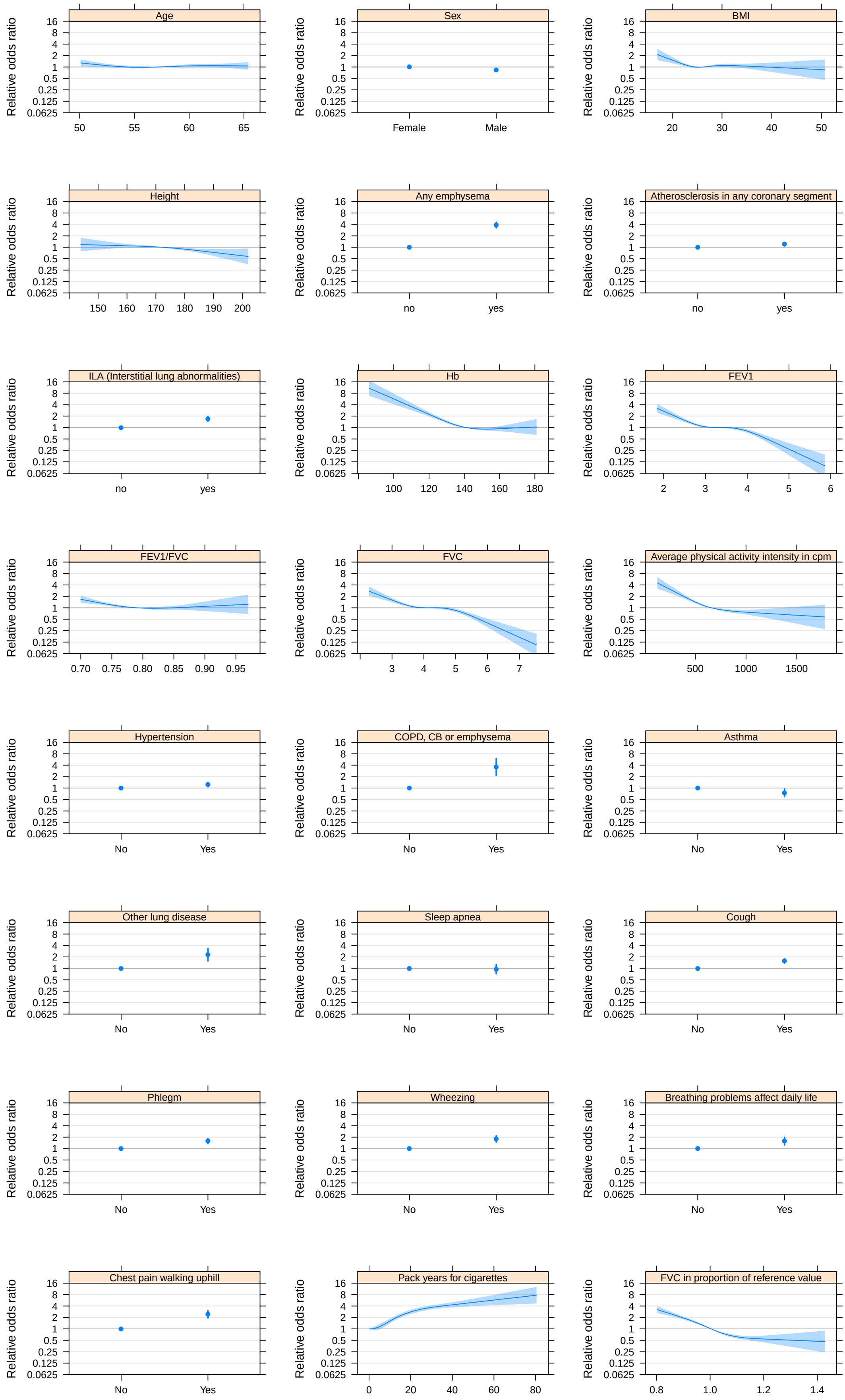

Supplementary Figure 1. Relative odds ratios for univariat analysis of main variables. The median is used as reference value for continous variables.

Var	N	From	To	OR	Chi2−df	P	P−nonlinear
Pack years for cigarettes	24261	0	10	1.67 (1.43−1.95)	286.24	<0.001	<0.001
Any emphysema	24261	No	Yes	2.78 (2.27−3.40)	97.74	<0.001	
FVC	24261	3.51	4.95	5.79 (3.89−8.60)	89.34	<0.001	0.004
ILA (Interstitial lung abnormalities)	24261	No	Yes	1.42 (1.21−1.68)	16.86	<0.001	
Age	24261	53.5	61	1.11 (0.93−1.32)	13.75	<0.001	<0.001
Chest pain walking uphill	24261	No	Yes	1.59 (1.23−2.05)	11.64	<0.001	
Cough	24261	No	Yes	1.29 (1.09−1.52)	7.89	0.003	
Breathing problems affect daily life	24261	No	Yes	1.39 (1.07−1.82)	4.90	0.015	
Other lung disease	24261	No	Yes	1.75 (1.11−2.76)	4.80	0.016	
Atherosclerosis in any coronary segment	24261	No	Yes	1.16 (1.01−1.33)	3.61	0.032	
Wheezing	24261	No	Yes	1.22 (0.96−1.56)	1.63	0.105	
Hypertension	24261	No	Yes	1.08 (0.93−1.24)	−0.02	0.323	
Phlegm	24261	No	Yes	1.07 (0.87−1.32)	−0.55	0.503	
COPD, CB or emphysema	24261	No	Yes	1.20 (0.65−2.20)	−0.67	0.568	
Sleep apnea	24261	No	Yes	0.83 (0.61−1.13)	0.42	0.234	
Sex	24261	Female	Male	0.71 (0.55−0.92)	5.97	0.008	
Asthma	24261	No	Yes	0.58 (0.44−0.76)	14.54	<0.001	
Height, cm	24261	165	179	0.34 (0.25−0.45)	83.97	<0.001	0.884
Average physical activity intensity in cpm	24261	535	796	0.66 (0.57−0.78)	108.53	<0.001	0.001
BMI, kg/m^2	24261	23.9	29.2	0.66 (0.57−0.77)	128.42	<0.001	0.354
Hb, g/L	24261	133	149	0.54 (0.45−0.64)	207.35	<0.001	<0.001
VA (z−score)	24261	−0.58	0.63	0.16 (0.13−0.20)	704.67	<0.001	0.013

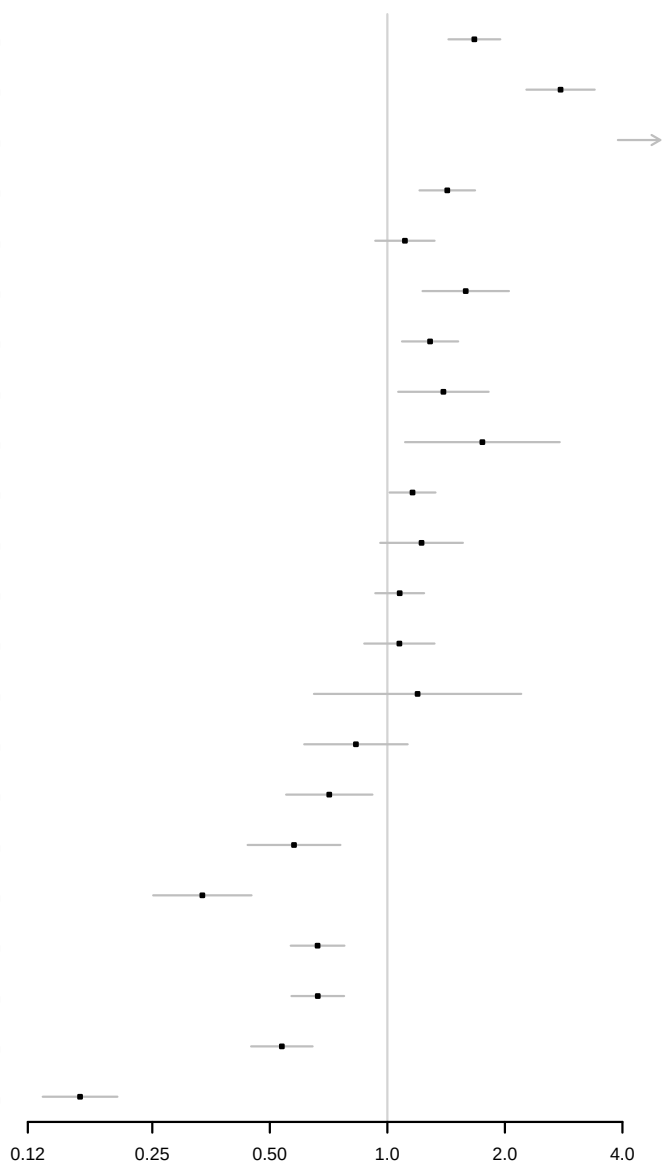

Supplementary Figure 2. Forestplot showing odds ratios for multivariable analysis of main variables (excluding FEV1 and FEV1/FVC but including VA), FVC in absolute terms. Odds ratios for the continuous variables are for the change between first and third quartile, inter quartile range (IQR).

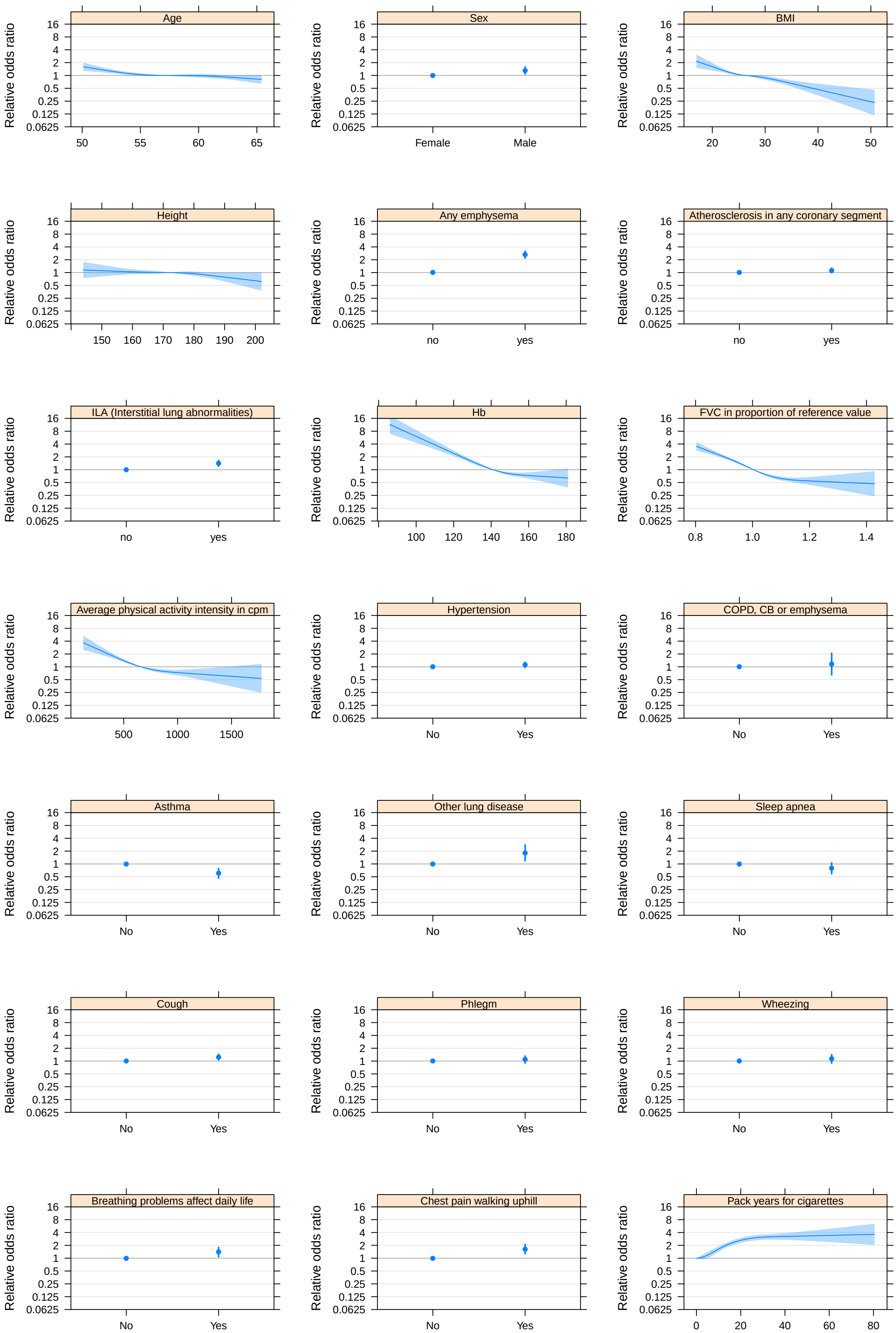

Supplementary figure 3. Log odds ratios for analysis of main variables in multivariable model. The median is used as reference value for continuous variables.

Var	N	From	To	HR	Chi2−df	P	P−nonlinear
Pack years for cigarettes	20177	0	10	1.63 (1.38−1.93)	187.33	<0.001	<0.001
Any emphysema	20177	No	Yes	2.50 (2.01−3.12)	65.53	<0.001	
ILA (Interstitial lung abnormalities)	20177	No	Yes	1.37 (1.14−1.64)	10.60	<0.001	
Chest pain walking uphill	20177	No	Yes	1.56 (1.16−2.10)	7.51	0.004	
Other lung disease	20177	No	Yes	1.79 (1.10−2.92)	4.45	0.020	
Cough	20177	No	Yes	1.22 (1.02−1.45)	3.85	0.028	
Sex	20177	Female	Male	1.28 (1.02−1.60)	3.69	0.030	
Wheezing	20177	No	Yes	1.22 (0.93−1.58)	1.09	0.149	
Atherosclerosis in any coronary segment	20177	No	Yes	1.10 (0.95−1.26)	0.71	0.191	
Phlegm	20177	No	Yes	1.14 (0.92−1.42)	0.46	0.227	
Hypertension	20177	No	Yes	1.09 (0.93−1.28)	0.15	0.284	
Breathing problems affect daily life	20177	No	Yes	1.18 (0.87−1.61)	0.12	0.289	
COPD, CB or emphysema	20177	No	Yes	1.27 (0.67−2.39)	−0.45	0.458	
Sleep apnea	20177	No	Yes	0.75 (0.53−1.05)	1.76	0.097	
Height	20177	165	179	0.94 (0.75−1.18)	3.71	0.082	0.571
Asthma	20177	No	Yes	0.62 (0.46−0.84)	8.48	0.002	
Age	20177	53.5	61	0.85 (0.71−1.02)	20.25	<0.001	0.232
BMI	20177	23.9	29.2	0.79 (0.67−0.94)	30.12	<0.001	0.921
Average physical activity intensity in cpm	20177	535	796	0.63 (0.53−0.75)	78.06	<0.001	0.007
Hb	20177	133	149	0.62 (0.51−0.76)	93.36	<0.001	0.012
FVC in proportion of reference value*	20177	0.93	1.08	0.38 (0.32−0.46)	272.96	<0.001	0.001

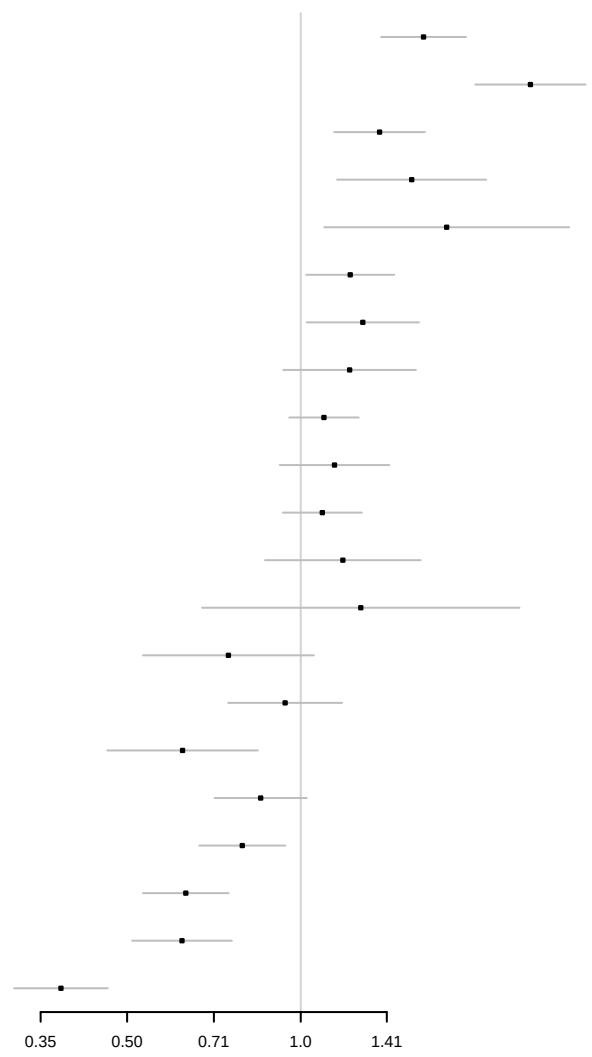

Supplementary Figure 4. Complete case Forest plot showing odds ratios for multivariable analysis of main variables. Odds ratios for the continous variables are for the change between first and third quartile, inter quartile range (IQR). ***Indicates SCAPIS reference equations (16)

Var	N	From	To	OR	Chi2−df	P	P−nonlinear
Pack years for cigarettes	24261	0	10	1.56 (1.34−1.81)	228.73	<0.001	<0.001
Any emphysema	24261	No	Yes	2.56 (2.12−3.11)	91.65	<0.001	
Height, cm	24261	165	179	2.18 (1.73−2.76)	85.68	<0.001	0.518
Sex	24261	Female	Male	2.45 (1.97−3.05)	64.09	<0.001	
Chest pain walking uphill	24261	No	Yes	1.70 (1.33−2.17)	16.68	<0.001	
ILA (Interstitial lung abnormalities)	24261	No	Yes	1.33 (1.14−1.55)	11.62	<0.001	
Breathing problems affect daily life	24261	No	Yes	1.41 (1.10−1.82)	6.11	0.008	
Other lung disease	24261	No	Yes	1.76 (1.15−2.69)	5.75	0.009	
Cough	24261	No	Yes	1.22 (1.04−1.42)	5.13	0.013	
Wheezing	24261	No	Yes	1.15 (0.91−1.45)	0.46	0.227	
Phlegm	24261	No	Yes	1.09 (0.90−1.32)	−0.24	0.382	
COPD, CB or emphysema	24261	No	Yes	1.17 (0.65−2.09)	−0.72	0.597	
Hypertension	24261	No	Yes	1.03 (0.90−1.18)	−0.83	0.679	
Atherosclerosis in any coronary segment	24261	No	Yes	1.01 (0.89−1.14)	−0.99	0.910	
Sleep apnea	24261	No	Yes	0.76 (0.57−1.02)	2.33	0.068	
Asthma	24261	No	Yes	0.62 (0.48−0.81)	11.80	<0.001	
BMI, kg/m^2	24261	23.9	29.2	0.84 (0.72−0.97)	45.23	<0.001	0.202
Average physical activity intensity in cpm	24261	535	796	0.66 (0.56−0.77)	108.42	<0.001	0.001
Hb, g/L	24261	133	149	0.61 (0.52−0.73)	162.56	<0.001	<0.001
FVC, L	24261	3.51	4.95	0.24 (0.19−0.32)	237.72	<0.001	0.012

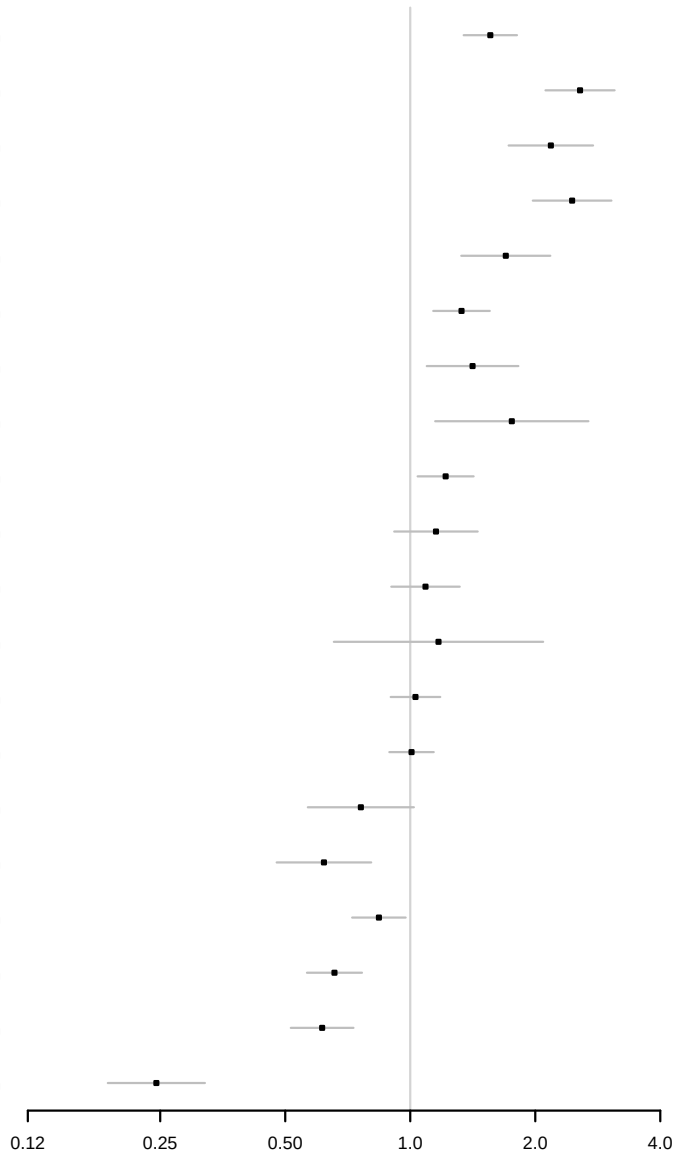

Supplementary Figure 5. Forestplot showing odds ratios for multivariable analysis of main variables (excluding FEV1 and FEV1/FVC and age), FVC in absolute terms. Odds ratios for the continuous variables are for the change between first and third quartile, interquartile range (IQR).

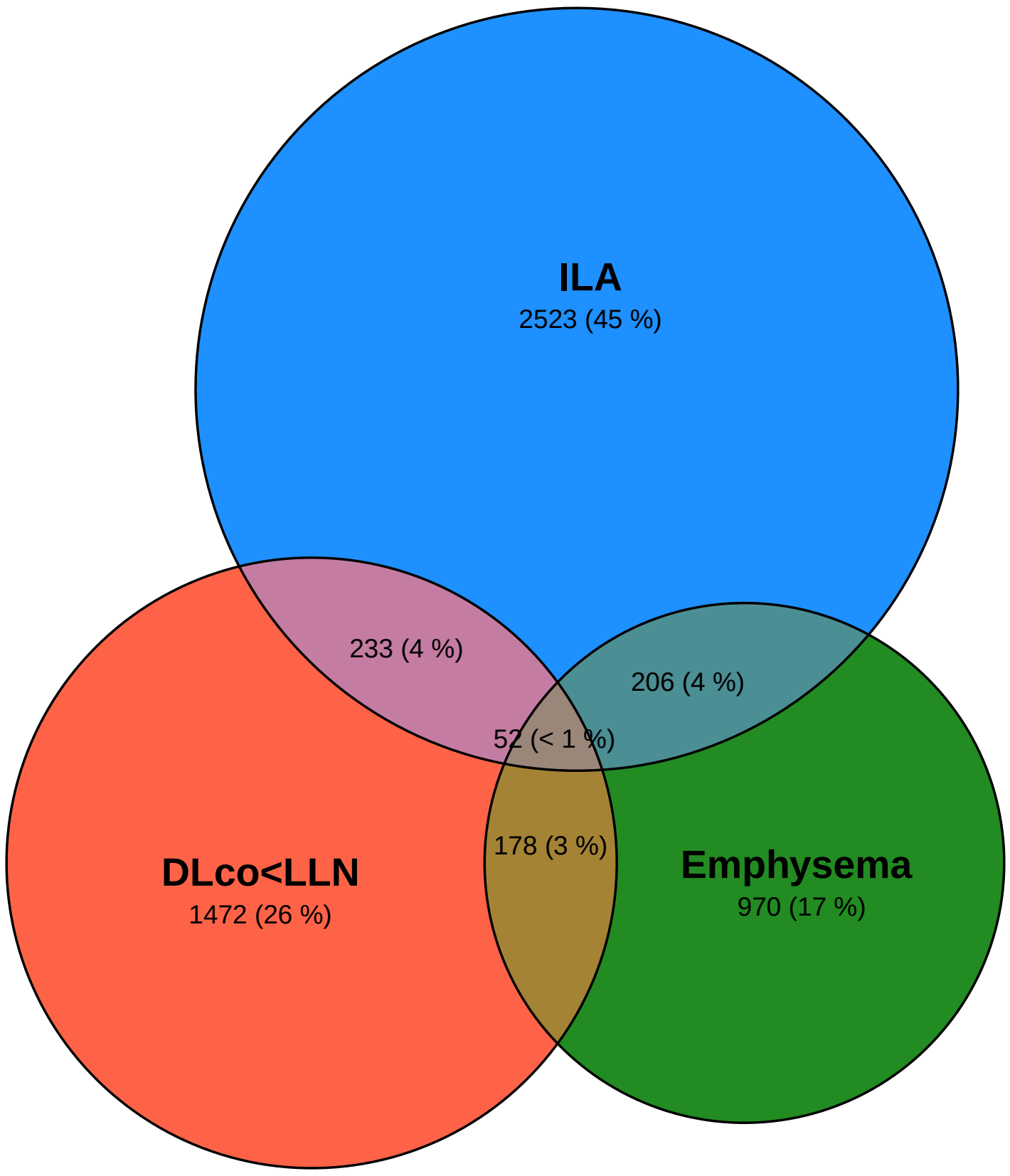

Supplementary figure 6. Venn diagram for ILA, D_{LCO}<LLN and emphysema.

	M	S	L
	exp(m0 + m1*ln(Height) -m2*ln(Age)<br>+ MSpline)	exp(s0 s1*ln(Age)<br>+ SSpline)	l

S = coefficient of variation
L = skewness

z-score = ((measured/M)^L - 1) / (L*S)
Proportion of predicted = measured/M

The parameters for

PostVA_M	PostVA_F	
-9,1642	-8,981	m0
2,2136	2,1402	m1
-0,0971	-0,0786	m2
-3,7557	-2,7434	s0
0,3906	0,1335	s1
0,8633	0,6301	l

Supplementary figure 7. The reference equation for VA used for calculating **%** of predicted and **Z**-score for VA
