# Supplementary material for: Isolated, reduced diffusing capacity for carbon monoxide in the population – relations to symptoms, smoking and comorbidities
Source: BMC Pulm Med. 2026 Aug 31;26:387. doi: 10.1186/s12890-026-04662-9 (PMC13531966; doi:10.1186/s12890-026-04662-9)
Supplement: Supplementary file 2 [file 12890_2026_4662_MOESM3_ESM.docx]

STROBE Statement—checklist of items that should be included in reports of observational studies

|  | Item No. | Recommendation | Page  No. | Relevant text from manuscript |
| --- | --- | --- | --- | --- |
| **Title and abstract** | 1 | (*a*) Indicate the study’s design with a commonly used term in the title or the abstract | 3 | Indicated in the abstract |
|  |  | (*b*) Provide in the abstract an informative and balanced summary of what was done and what was found | 3 | Described in the abstract |
| Introduction | | | |  |
| Background/rationale | 2 | Explain the scientific background and rationale for the investigation being reported | 5 | Section Introduction |
| Objectives | 3 | State specific objectives, including any prespecified hypotheses | 5 | Section Introduction |
| Methods | | | |  |
| Study design | 4 | Present key elements of study design early in the paper | 6-8 | Section Methods |
| Setting | 5 | Describe the setting, locations, and relevant dates, including periods of recruitment, exposure, follow-up, and data collection | 6 | Sub-section Subjects in Methods |
| Participants | 6 | (*a*) *Cohort study*—Give the eligibility criteria, and the sources and methods of selection of participants. Describe methods of follow-up  *Case-control study*—Give the eligibility criteria, and the sources and methods of case ascertainment and control selection. Give the rationale for the choice of cases and controls  ***Cross-sectional study*—Give the eligibility criteria, and the sources and methods of selection of participants** | 6 | Sub-section Subjects in Methods + Figure 1 |
|  |  | (*b*) *Cohort study*—For matched studies, give matching criteria and number of exposed and unexposed  *Case-control study*—For matched studies, give matching criteria and the number of controls per case |  |  |
| Variables | 7 | Clearly define all outcomes, exposures, predictors, potential confounders, and effect modifiers. Give diagnostic criteria, if applicable | 7-9 | Sub-section Statistics in Methods |
| Data sources/ measurement | 8* | For each variable of interest, give sources of data and details of methods of assessment (measurement). Describe comparability of assessment methods if there is more than one group | 7-9 | Section Methods |
| Bias | 9 | Describe any efforts to address potential sources of bias | 8 | Sub-section Statistics - Missing data in Methods |
| Study size | 10 | Explain how the study size was arrived at | 6 | Sub-section Subjects in Methods |

Continued on next page

| Quantitative variables | 11 | Explain how quantitative variables were handled in the analyses. If applicable, describe which groupings were chosen and why | 7-9 | Sub-section Statistics in Methods |
| --- | --- | --- | --- | --- |
| Statistical methods | 12 | (*a*) Describe all statistical methods, including those used to control for confounding | 7-9 | Sub-section Statistics in Methods |
|  |  | (*b*) Describe any methods used to examine subgroups and interactions | 7-9 | Sub-section Statistics in Methods |
|  |  | (*c*) Explain how missing data were addressed | 7-9 | Sub-section Statistics in Methods |
|  |  | (*d*) *Cohort study*—If applicable, explain how loss to follow-up was addressed  *Case-control study*—If applicable, explain how matching of cases and controls was addressed  *Cross-sectional study*—If applicable, describe analytical methods taking account of sampling strategy |  |  |
|  |  | (*e*) Describe any sensitivity analyses | 7-9 | Sub-section Statistics in Methods |
| Results | | | | |
| Participants | 13* | (a) Report numbers of individuals at each stage of study—eg numbers potentially eligible, examined for eligibility, confirmed eligible, included in the study, completing follow-up, and analysed | 6 | Sub-section Subjects in Methods + Figure 1 |
|  |  | (b) Give reasons for non-participation at each stage | 6 | Sub-section Subjects in Methods + Figure 1 |
|  |  | (c) Consider use of a flow diagram | 6 | Figure 1 |
| Descriptive data | 14* | (a) Give characteristics of study participants (eg demographic, clinical, social) and information on exposures and potential confounders | 10 | Sub-section Study population characteristics in Results + Table 1 |
|  |  | (b) Indicate number of participants with missing data for each variable of interest | 10 | Supplementary Tables 1-2 |
|  |  | (c) *Cohort study*—Summarise follow-up time (eg, average and total amount) |  |  |
| Outcome data | 15* | *Cohort study*—Report numbers of outcome events or summary measures over time |  |  |
|  |  | *Case-control study—*Report numbers in each exposure category, or summary measures of exposure |  |  |
|  |  | *Cross-sectional study—*Report numbers of outcome events or summary measures | 10 | Sub-section Study population descriptives in Results + Table 1 |
| Main results | 16 | (*a*) Give unadjusted estimates and, if applicable, confounder-adjusted estimates and their precision (eg, 95% confidence interval). Make clear which confounders were adjusted for and why they were included | 10 | Sub-section Factors associated with reduced DLCO in Results + Figures 2-3 |
|  |  | (*b*) Report category boundaries when continuous variables were categorized | 10 | Sub-section Factors associated with reduced DLCO in Results + Figures 2-3 |
|  |  | (*c*) If relevant, consider translating estimates of relative risk into absolute risk for a meaningful time period | N/A |  |

Continued on next page

| Other analyses | 17 | Report other analyses done—eg analyses of subgroups and interactions, and sensitivity analyses | 10-11 | Sub-section Factors associated with isolated reduced DLCO, Interactions with smoking status and Overlap and interactions between ILA, radiological emphysema and reduced DLCO in Results + Figure 4 + Supplementary Figures 1-4 |
| --- | --- | --- | --- | --- |
| Discussion | | | | |
| Key results | 18 | Summarise key results with reference to study objectives | 12 | First paragraph of Discussion |
| Limitations | 19 | Discuss limitations of the study, taking into account sources of potential bias or imprecision. Discuss both direction and magnitude of any potential bias | 15 | Section Study strengths and limitations in Discussion |
| Interpretation | 20 | Give a cautious overall interpretation of results considering objectives, limitations, multiplicity of analyses, results from similar studies, and other relevant evidence | 12-16 | Covered from varies point of views in the sections of the discussion |
| Generalisability | 21 | Discuss the generalisability (external validity) of the study results | 15 | Section Study strengths and limitations in Discussion |
| Other information | |  | | |
| Funding | 22 | Give the source of funding and the role of the funders for the present study and, if applicable, for the original study on which the present article is based | 18 | Section funding |

*Give information separately for cases and controls in case-control studies and, if applicable, for exposed and unexposed groups in cohort and cross-sectional studies.

**Note:** An Explanation and Elaboration article discusses each checklist item and gives methodological background and published examples of transparent reporting. The STROBE checklist is best used in conjunction with this article (freely available on the Web sites of PLoS Medicine at http://www.plosmedicine.org/, Annals of Internal Medicine at http://www.annals.org/, and Epidemiology at http://www.epidem.com/). Information on the STROBE Initiative is available at www.strobe-statement.org.
